# A bivalent COVID-19 mRNA vaccine elicited broad immune responses and protection against Omicron subvariants infection

**DOI:** 10.1038/s41541-025-01062-8

**Published:** 2025-01-10

**Authors:** Jun Liu, Li Wang, Alexandra Kurtesi, Patrick Budylowski, Kyle G. Potts, Haritha Menon, Yilin Tan, Philip Samaan, Xinan Liu, Yisen Wang, Queenie Hu, Reuben Samson, Freda Qi, Danyel Evseev, Cini John, Kristofor K. Ellestad, Yue Fan, Frans Budiman, Ellaine Riczly Tohan, Suji Udayakumar, Jennifer Yang, Eric G. Marcusson, Anne-Claude Gingras, Douglas J. Mahoney, Mario A. Ostrowski, Natalia Martin-Orozco

**Affiliations:** 1 Providence Therapeutics Holdings, Inc., Calgary, Canada; 2 Everest Medicines, Shanghai, China; 3 Department of Molecular Genetics, University of Toronto, Toronto, Canada; 4 Lunenfeld-Tanenbaum Research Institute at Mount Sinai Hospital, Sinai Health System, Toronto, Canada; 5 Department of Medicine, University of Toronto, Toronto, Canada; 6 Institute of Medical Science, University of Toronto, Toronto, Canada; 7 Riddell Center for Cancer Immunotherapy, Cumming School of Medicine, University of Calgary, Calgary, Canada; 8 Arnie Charbonneau Cancer Institute, Cumming School of Medicine, University of Calgary, Calgary, Canada; 9 Alberta Children’s Hospital Research Institute, Cumming School of Medicine, University of Calgary, Calgary, Canada; 10 Molecular Biology and Microbiology, Immunology, and Infectious Diseases, Cumming School of Medicine, University of Calgary, Calgary, Canada; 11 Department of Laboratory Medicine and Pathobiology, University of Toronto, Toronto, Canada; 12 Department of Immunology, University of Toronto, Toronto, Canada; 13 Keenan Research Centre for Biomedical Science of St. Michael’s Hospital, Unity Health Toronto, Toronto, Canada

**Keywords:** RNA vaccines, RNA vaccines

## Abstract

Continuously emerging SARS-CoV-2 Omicron subvariants pose a threat thwarting the effectiveness of approved COVID-19 vaccines. Especially, the protection breadth and degree of these vaccines against antigenically distant Omicron subvariants is unclear. Here, we report the immunogenicity and efficacy of a bivalent mRNA vaccine, PTX-COVID19-M1.2 (M1.2), which encodes native spike proteins from Wuhan-Hu-1 (D614G) and Omicron BA.2.12.1, in mouse and hamster models. Both primary series and booster vaccination using M1.2 elicited potent and broad nAbs against Wuhan-Hu-1 (D614G) and some Omicron subvariants. Strong spike-specific T cell responses against Wuhan-Hu-1 and Omicron subvariants, including JN.1, were also induced. Vaccination with M1.2 protected animals from Wuhan-Hu-1 and multiple Omicron subvariants challenges. Interestingly, protection against XBB.1.5 lung infection did not correlate with nAb levels. These results indicate that M1.2 generated a broadly protective immune response against antigenically distant Omicron subvariants, and spike-specific T cells probably contributed to the breadth of the protection.

## Introduction

mRNA vaccines have revolutionized vaccine development and played a critical role in fighting the Coronavirus disease 2019 (COVID-19) pandemic. Primary series (two-dose) immunization with the first two approved COVID-19 mRNA vaccines showed high efficacy and safety in clinical trials and subsequent real-world administration^1–4^. However, rapid waning of the vaccine-induced nAb response and the continuous emergence of Severe acute respiratory syndrome coronavirus 2 (SARS-CoV-2) variants of concern (VOCs), especially the advent of the Omicron variants, significantly eroded the effectiveness of these prototype mRNA vaccines in preventing infection^5,6^. Despite this, the effectiveness of the mRNA vaccines in protection against severe disease and death was more durable and resilient to the spike mutations occurring in VOCs^2,5–10^. Booster doses (3^rd^ or more doses) of the mRNA vaccines can strengthen the weakened nAbs and regain vaccine effectiveness (VE) against infection, especially when using updated spike sequences from VOCs^11–13^. In this regard, bivalent mRNA vaccines composed of ancestral Wuhan-Hu-1 spike used in the monovalent prototype mRNA vaccines and various VOC spikes (especially the spike from the circulating Omicron subvariants) were demonstrated in both animals and human subjects to enhance the magnitude and breadth of nAbs, which correlated with improved VE^14,15^. Previous studies also showed that the VOC spikes in the bivalent vaccines need to be homologous or antigenically closely matched to elicit high level nAbs against the target VOCs^16–20^. However, it is uncertain if the bivalent vaccines can provide adequate protection against antigenically distant SARS-CoV-2 VOCs in the context of low or negligible cross-neutralizing Abs. Given the difficulty in matching vaccine spike protein to contemporary VOCs, a bivalent mRNA vaccine or other forms of spike-based vaccines that can provide cross-protection against antigenically distant VOCs to reduce morbidity and mortality and stifle virus spreading are desirable. It is thus important to investigate the breadth of the protection afforded by bivalent mRNA vaccines and elucidate the underlying mechanisms.

As in many viral infections, serum nAbs are regarded as the main immune mechanism underlying vaccine-induced protection against SARS-CoV-2 infection^21,22^. Indeed, nAb titer has been recommended as an immune protection correlate in vaccinated or post-infection human subjects^21,23^. Recent studies also reveal the importance of T cells in vaccine-induced protection against SARS-CoV-2 infection and disease. For example, mRNA vaccines can still provide considerable protection against COVID-19 hospitalization and death in the absence of sufficient nAbs, and the protection was putatively mediated by spike-specific T cells^6–8^. Adoptive transfer of T cells decreased SARS-CoV-2 replication, alleviated the clinical manifestations in animals and promoted recovery in severe COVID-19 patients^24,25^. Spike-specific T cells emerged earlier than nAbs in humans after prime mRNA vaccination, coinciding with the early onset of the protection afforded by the vaccines^26,27^. More recently, spike-specific memory T cells raised by monovalent prototype mRNA vaccines were found to respond earlier than memory B cells and cross-neutralizing Ab in Omicron breakthrough infections (BTI), and the rapid responding spike-specific memory CD8 + T cells after VOC Delta or Omicron BTI correlated with faster viral clearance in human subjects previously vaccinated with Wuhan-Hu-1 spike-based vaccine^28,29^. Compared to nAbs, spike-specific T cell response elicited by prototype mRNA vaccines or previous infections is more durable and less affected by the mutations in the spike protein^30–34^. Besides T cells, non-neutralizing Abs may also participate in clearing virus-infected cells^20^. Taken together, these recent findings suggest a bivalent mRNA vaccine could provide broad protection against circulating and emerging VOCs by immune mechanisms other than nAbs, such as T cells and non-neutralizing Abs.

Immune imprinting defines the phenomenon that memory immune responses generated from a host’s previous exposure to a pathogen modulate the induction of immune responses when subsequently encountering variants of the same pathogen^35^. Immune imprinting may constitute a major hurdle for booster vaccination’s effectiveness against SARS-CoV-2 by suppressing the induction of nAbs against emerging VOCs^16,20,36^. Booster doses with bivalent vaccine preferentially increased the magnitude of nAbs against Wuhan-Hu-1 over VOCs^15,19,20,37,38^. Indeed, monovalent mRNA vaccines based on Omicron XBB.1.5 spike were recommended for the 2023 fall season partly due to the concern of immune imprinting^39,40^. However, even the monovalent Omicron spike vaccines still favored boosting nAbs against Wuhan-Hu-1 over generating nAbs against Omicron variants in humans previously vaccinated with Wuhan-Hu-1 spike-based vaccines^19^. It is less clear if immune imprinting also affects spike-specific T cells to reduce the effectiveness of booster vaccinations^41^.

We previously reported the results from preclinical studies and clinical trials of our prototype COVID-19 mRNA vaccine, PTX-COVID19-B^42–44^. PTX-COVID19-B encodes native full-length spike sequence from Wuhan-Hu-1 with D614G mutation, and demonstrated high immunogenicity, efficacy, and safety in animal models and human subjects. Here, we report our studies on the immunogenicity and efficacy of our bivalent COVID-19 mRNA vaccine, PTX-COVID19-M1.2 (designated as M1.2 hereafter in this paper), in animals. We found M1.2 elicited broad nAb and T cell response and protected mice and hamsters against multiple Omicron subvariants, including Omicron XBB.1.5 in the absence of high titer nAbs. Furthermore, we found little immune imprinting on spike-specific T cells in mice receiving prototype mRNA vaccine as primary series followed by bivalent mRNA vaccine booster.

## Results

### M1.2 elicited a broad nAb response against SARS-CoV-2 ancestral strain and Omicron subvariants when used in primary series vaccination

**Fig. 1:**
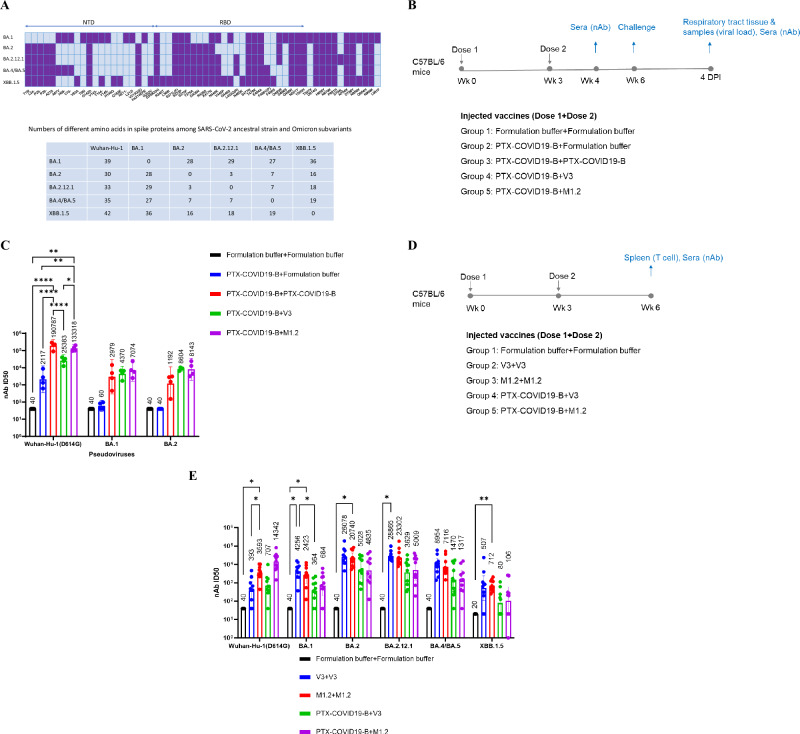
Bivalent mRNA vaccine M1.2 elicit potent and broad nAbs. **A** Schematic representation of spike protein mutations in Omicron subvariants. Top panel shows locations of mutated amino acids compared to Wuhan-Hu-1 spike, dark purple indicating presence of mutation and light purple indicating absence of mutation. NTD denotes N-terminal domain of spike, and RBD denotes receptor binding domain of spike. Lower panel shows the number of different amino acids of the pair-wise compared Wuhan-Hu-1 and Omicron subvariant spikes. **B** Immunogenicity and challenge experiment schedule. Six- to eight-week-old female C57BL/6 mice (*n* = 10) were immunized twice i.m. with 10 µg mRNA vaccines or control formulation buffer and challenged with Omicron BA.1 three weeks post 2^nd^ vaccination. **C** nAbs of mice sera. Sera from mice in **B** were collected at 1 week post 2^nd^ vaccination (week 4). For each group, every 2**–**3 mice sera were pooled (4 pools per group) to be used in nAb pseudovirus assay. Columns and error bars indicate geometric mean±95% confidence interval of the nAb ID_50_ (*n* = 4). Each dot represents an individual pool. The numbers above columns indicate geometric mean values of the nAb ID_50_. LLOQ of the nAb assay is 80, and when serum nAb ID_50_ values were below LLOQ, half of the LLOQ values (40) were assigned to the sera. **D** Immunogenicity experiment schedule. Six- to eight-week-old female C57BL/6 mice (*n* = 10 except *n* = 8 for Formulation buffer control group) were immunized twice i.m. with 10 µg mRNA vaccines or control Formulation buffer. Sera and spleens were collected 3 weeks post 2^nd^ vaccination. **E** nAbs of mice sera. Sera from mice in **D** were collected at 3 weeks post 2^nd^ vaccination (week 6). Individual mouse sera were used in nAb pseudovirus assay. Columns and error bars indicate geometric mean±95% confidence interval of the nAb ID_50_ (*n* = 10 except *n* = 8 for Formulation buffer control group). Each dot represents an individual mouse. The numbers above columns indicate geometric mean values of the nAb ID_50_. LLOQ of the nAb assay is 80 except 40 for XBB.1.5 pseudovirus. When serum nAb ID_50_ values were below LLOQ, half of the LLOQ values (40 except 20 for XBB.1.5 pseudovirus) were assigned to the sera. Two-way ANOVA followed by Tukey’s multiple comparison was used for statistical analysis. *P < 0.05; **P < 0.01; ***P < 0.001; ****P < 0.0001.

To adapt to the continuing emergence of SARS-CoV-2 variants, especially the Omicron subvariants, we constructed a bivalent spike-based mRNA vaccine, M1.2, which is a 1:1 mass ratio mixture of the mRNA in our prototype mRNA vaccine, PTX-COVID19-B^42^, encoding the Wuhan-Hu-1 spike protein with D614G mutation, and an mRNA encoding Omicron BA.2.12.1 spike. BA.2.12.1 was the predominant Omicron subvariant when this study was initiated. Monovalent BA.2.12.1 spike mRNA vaccine, named PTX-COVID19-V3 (designated as V3 hereafter in this paper), was also constructed as a control. The spike mutations of Omicron subvariants, including BA.1, BA.2, BA.2.12.1, BA.4/BA.5, and XBB.1.5, compared to the ancestral Wuhan-Hu-1, are shown in Fig. 1A. As shown in Fig. 1B, C57BL/6 mice (*n* = 10) were intramuscularly vaccinated twice with the monovalent prototype spike vaccine PTX-COVID19-B, or, primed with PTX-COVID19-B and then followed by a boost using either V3 or bivalent M1.2 or control formulation buffer. An additional control group of mice received two injections of the formulation buffer. Doses of the mRNA vaccines were 10 µg per mouse per vaccination. One week post the 2^nd^ vaccination, serum was collected from each mouse and every 2 or 3 individual mouse sera were pooled together to form 4 separate sera pools for each treatment group. nAbs in these pooled sera were measured against Wuhan-Hu-1 with the spike D614G mutation (Wuhan-Hu-1 (D614G)), Omicron BA.1 and BA.2 using a pseudovirus neutralization assay as reported before^42^.

We first compared the nAb response against Wuhan-Hu-1 (D614G) in the sera from the vaccinated mice. As shown in Fig. 1C, no nAbs against Wuhan-Hu-1 (D614G) pseudovirus were detected in the sera of control mice receiving two injections of the formulation buffer, and modest levels of nAbs were detected in control mice primed with one dose of PTX-COVID19-B and boosted with formulation buffer. In contrast, sera from all mice receiving two doses of PTX-COVID19 vaccines exhibited potent nAb activity against the Wuhan-Hu-1 (D614G) pseudovirus. Among them, two doses of PTX-COVID19-B vaccination elicited the highest nAbs against Wuhan-Hu-1 (D614G), which are significantly higher than that induced by PTX-COVID19-B prime plus monovalent V3 boost (*P* < 0.0001). PTX-COVID19-B prime and M1.2 boost-induced nAb titer is comparable to two doses of PTX-COVID19-B, and significantly higher than PTX-COVID19-B prime plus monovalent V3 boost (*P* < 0.05). We then compared the nAbs against Omicron BA.1 and BA.2 pseudoviruses (Fig. 1C). Sera from the formulation buffer treated control mice did not show any nAb activity against BA.1 or BA.2. For both BA.1 and BA.2, PTX-COVID19-B prime boosted by bivalent M1.2 or monovalent V3 induced higher nAbs than that elicited by two doses of PTX-COVID19-B (Fig. 1C), though this did not reach statistical significance. Additionally, compared to V3 boost, M1.2 boost elicited slightly higher nAbs against BA.1, which is not statistically significant, and similar levels of nAbs against BA.2. These results demonstrate that bivalent M1.2 can elicit potent nAbs against both SARS-CoV-2 ancestral strain Wuhan-Hu-1 (D614G) and Omicron subvariants BA.1 and BA.2 (Fig. 1C and Table 1).

**Table 1:** Geometric mean ratio of nAb ID50 titers in vaccinated mice sera

Vaccine regimens ^a^	Pseudoviruses		
	Wuhan-Hu-1 (D614G)	Omicron BA.1	Omicron BA.2
PTX-COVID19-B + PTX-COVID19-B ^b^	1	1	1
PTX-COVID19-B + Formulation buffer	0.01	0.004	0.001
PTX-COVID19-B + V3	0.13	1.47	7.22
PTX-COVID19-B + M1.2	0.7	2.37	6.83

^a^ Prime vaccine + boost vaccine. ^b^Reference group.

We corroborated that M1.2 could elicit broad and potent nAbs in a separate experiment where different groups of mice received either two doses (in prime and boost vaccination) or one dose (as a boost following PTX-COVID19-B prime) of M1.2 or V3 (Fig. 1D,E). Sera were isolated from these mice 3 weeks post 2^nd^ vaccination and the nAb response in the individual mouse serum (*n* = 10) was tested against Wuhan-Hu-1 (D614G) and multiple Omicron subvariants, including BA.1, BA.2, BA.2.12.1, BA.4/BA.5, and XBB.1.5 using the same pseudovirus neutralizing assay. As shown in Fig. 1E, bivalent M1.2 always elicited higher nAbs against Wuhan-Hu-1 (D614G) than monovalent V3, whether in the two dose (prime-boost) regimen (*P* < 0.05) or in the 2^nd^ dose-only (boost) regimen (*P* = 0.097). M1.2 also induced nAbs against Omicron subvariants at similar level to monovalent V3, indicating that M1.2 can be used as a booster vaccine. In addition, two doses of PTX-COVID19-M1.2 was the only regimen that generated significantly higher titer of nAbs against XBB.1.5 than the formulation buffer control (*P* < 0.01). We also noticed that two doses of M1.2 induced higher nAb titers against BA.2, BA.2.12.1, and BA.4/BA.5 pseudoviruses than against Wuhan-Hu-1 (D614G) pseudovirus (GMT: 20740, 23302, 7116, and 3593, respectively), although these are not statistically significant. Taken together, these results indicate that the bivalent M1.2 used in primary series vaccination can elicit potent and broad nAb responses against SARS-CoV-2 ancestral strain and Omicron subvariants.

### M1.2 elicited cross-reactive T cell responses against SARS-CoV-2

**Fig. 2:**
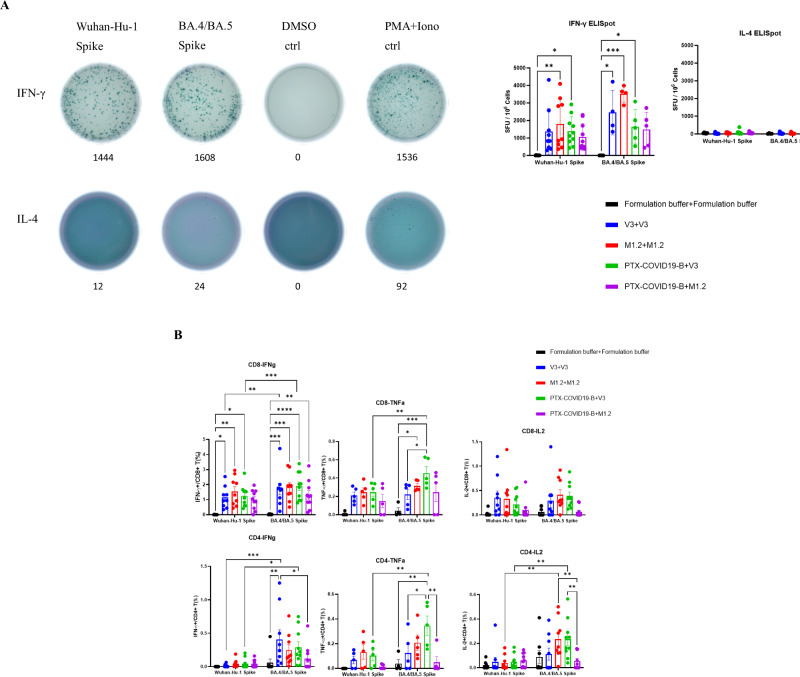
Bivalent mRNA vaccine M1.2 elicit potent and broad T cell response. **A** ELISpot results. Splenocytes from the mice in Fig. 1D were stimulated with peptide pools from Wuhan-Hu-1 or Omicron BA.4/BA.5 spike proteins. Representative spot graphs from one mouse are shown in the upper panel. Numbers below each graph are the spot forming units (SFU) per million cells for the graph. Columns and error bars indicate mean± S.E.M. of SFU per million input cells (n = 4-9). Each dot represents an individual mouse. **B** T cell ICS results. Splenocytes from the mice in Fig. 1D were stimulated with peptide pools from Wuhan-Hu-1 or Omicron BA.4/BA.5 spike proteins. Column and error bars indicate mean± S.E.M. of percentage of cytokine positive cells in total CD4+ or CD8 + T cells (*n* = 8**–**10). Each dot represents an individual mouse. Two-way ANOVA followed by Tukey’s multiple comparison within same peptide stimulation or Šidák’s multiple comparison between different peptide stimulations was used for statistical analysis. *P < 0.05; **P < 0.01; ***P < 0.001; ****P < 0.0001.

T cells are thought to play a critical role in protecting against symptomatic COVID-19^28,32^. Furthermore, CD4 + T cells are pivotal in orchestrating high-quality humoral and cellular immune responses^27^. We therefore measured spike-specific T cell responses induced by bivalent M1.2 and compared it with that elicited by monovalent V3. Splenocytes from the vaccinated mice shown in Fig. 1D were collected at 3 weeks post 2^nd^ vaccination, stimulated ex vivo with either a Wuhan-Hu-1 spike peptide pool or Omicron BA.4/BA.5 spike peptide pool, and spike-specific T cell responses were quantified by ELISpot and intracellular cytokine flow cytometry. As shown in Fig. 2A, both monovalent V3 and bivalent M1.2 induced a spike-specific Th1-biased response. This potent induction of spike-specific Th1 cytokines by both monovalent V3 and bivalent M1.2 vaccines was also confirmed by flow cytometry results (Fig. 2B and Supplementary Fig. 1C), showing high percentage of spike-specific Th1 cytokine (IFN-γ/ TNF-α/IL-2)-producing CD4 + T and CD8 + T cells and low percentage of spike-specific Th2 cytokine (IL-4/IL-5)-producing T cells. This is consistent with our previous findings on T cell response induced by the prototype COVID-19 mRNA vaccine, PTX-COVID19-B^42^. Notably, Wuhan-Hu-1 and BA.4/BA.5 spike peptide pools ex vivo stimulation generated similar levels of cytokine production from the CD4+ and CD8 + T cells of the mice receiving bivalent M1.2 vaccine, except for IL-2-producing CD4 + T cells from the mice receiving two doses of M1.2, which was significantly more induced after BA.4/BA.5 spike peptide pool stimulation than Wuhan-Hu-1 spike (*P* < 0.01, Fig. 2B and Table 2). In contrast, in the mice receiving the monovalent V3 vaccine, the BA.4/BA.5 spike peptide pool stimulated more cytokine-producing T cells than the Wuhan-Hu-1 spike peptide pool, including IFN-γ-producing CD4+ and CD8 + T cells from mice receiving either one or two doses of V3, and TNF-α producing CD4+ and CD8 + T cells and IL-2 producing CD4 + T cells from mice receiving one dose of V3 (Fig. 2B, Table 2). Furthermore, comparison of T cell responses induced by bivalent M1.2 and monovalent V3 indicated that they were generally comparable except there were more IL-2-producing and TNF-α-producing CD4 + T cells in the mice receiving V3 boost than M1.2 boost when the splenocytes were stimulated with BA.4/BA.5 spike peptide pool (*P* < 0.01, Fig. 2B, Table 3). Taken together, these results indicate that the bivalent vaccine M1.2 induced a broader T cell response, which targeted both ancestral Wuhan-Hu-1 spike and Omicron BA.4/BA.5 spike, than the monovalent V3, which preferentially elicited T cell response against Omicron BA.4/BA.5.

**Table 2:** Vaccination regimens that generated cytokine-producing T cells statistically higher in magnitude against BA.4/BA.5 spike than against Wuhan-Hu-1 spike

Cytokine-producing T cells	Vaccine regimens ^#^	spike peptide pool used in stimulation	*P* value ^*^
IFN-γ-producing CD8 + T cells	V3 + V3	BA.4/BA.5 spike peptide pool *versus* Wuhan-Hu-1 spike peptide pool	0.0029
	PTX-COVID19-B + V3		0.0003
IFN-γ-producing CD4 + T cells	V3 + V3		0.0007
	PTX-COVID19-B + V3		0.0391
TNF-α-producing CD8 + T cells	PTX-COVID19-B + V3		0.0033
TNF-α-producing CD4 + T cells	PTX-COVID19-B + V3		0.0087
IL-2-producing CD4 + T cells	PTX-COVID19-B + V3		0.0022
	M1.2 + M1.2		0.0023

^#^Prime vaccine + boost vaccine. *Two-way ANOVA followed by Šidák’s multiple comparison test.

**Table 3:** Cytokine-producing T cells that were statistically different between V3 and M1.2 vaccination regimens

Cytokine-producing T cells	Vaccine regimens	spike peptide pool used in stimulation	*P* value^*^
IL-2-producing CD4 + T cells	PTX-COVID19-B + V3 *versus* PTX-COVID19-B + M1.2	BA.4/BA.5 spike peptide pool	0.0048
TNF-α-producing CD4 + T cells			0.0013

*Two-way ANOVA followed by Tukey’s multiple comparison test.

### M1.2 as the booster (3^rd^) dose enhanced immune response against SARS-CoV-2 Omicron subvariants

**Fig. 3:**
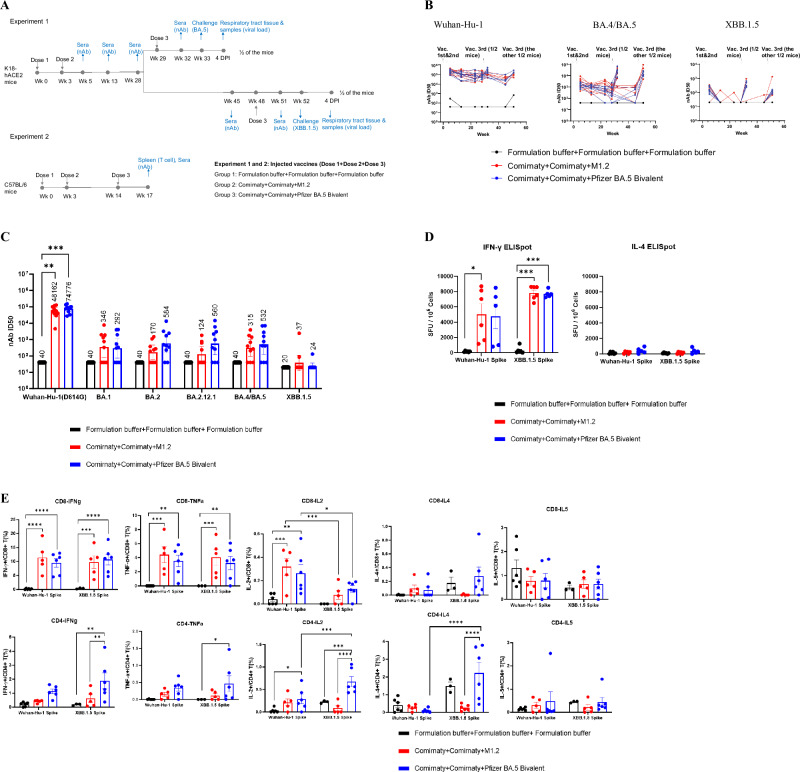
Booster dose of bivalent mRNA vaccine M1.2 elicit potent and broad nAb and T cell response. **A** Booster experiment schedules. For Experiment 1, six- to eight-week-old female K18-hACE2 mice (*n* = 10) were immunized three times intramuscularly (i.m.) with 5 µg mRNA vaccines or control Formulation buffer and challenged with Omicron BA.5 (*n* = 5) or XBB.1.5 (*n* = 4–5) three weeks post 3^rd^ vaccination. For Experiment 2, six- to eight-week-old female C57BL/6 mice (*n* = 10) were immunized three times i.m. with 5 µg mRNA vaccines or control Formulation buffer, and then sera and spleens were collected at 3 weeks post 3^rd^ vaccination. **B** Dynamic of nAbs in mice sera. Sera from mice in **A** Experiment 1 were collected at indicated time points and used in nAb pseudovirus assay. Each dot connected by a line indicates nAb ID_50_ of individual mouse serum. **C** nAbs of mice sera. Sera from mice in **A** Experiment 2 were collected at 3 weeks post 3^rd^ vaccination and used in nAb pseudovirus assay. Columns and error bars indicate nAb ID_50_ geometric means±95% confidence interval of individual mouse serum (*n* = 10). Each dot represents an individual mouse. LLOQ of the nAb assay is 80 except 40 for XBB.1.5 pseudovirus. When serum nAb ID_50_ values were below LLOQ, half of the LLOQ values (40 except 20 for XBB.1.5 pseudovirus) were assigned to the sera. The numbers on top of the columns are geometric mean values of the nAb ID_50_. **D** ELISpot results. Splenocytes from the mice in (A) Experiment 2 were stimulated with peptide pools from Wuhan-Hu-1 or Omicron XBB.1.5 spike proteins. Columns and error bars indicate mean± S.E.M. of spot forming units (SFU) per million input cells. Each dot represents an individual mouse. **E** T cell ICS results. Splenocytes from the mice in **A** Experiment 2 were stimulated with peptide pools from Wuhan-Hu-1 or XBB.1.5 spike proteins. Columns and error bars indicate mean± S.E.M. of percentage of cytokine positive cells in total CD4+ or CD8 + T cells (*n* = 3–6). Each dot represents an individual mouse. Two-way ANOVA followed by Tukey’s multiple comparison was used for statistical analysis of **C**. Two-way ANOVA followed by Tukey’s multiple comparison within same peptide stimulation or Šidák’s multiple comparison between different peptide stimulations was used for statistical analysis of **D** and **E**. *P < 0.05; **P < 0.01; ***P < 0.001; ****P < 0.0001.

Based on the immunogenicity of M1.2 in the primary series vaccination described above, we then tested if M1.2 could enhance the immune response induced by previous vaccinations with prototype vaccines. Two independent experiments were performed for this purpose (Fig. 3A). In Experiment 1, K18-hACE2 mice (*n* = 10 per group) were vaccinated twice with 5 µg monovalent Comirnaty (encoding ancestral strain spike only), and then received 5 µg M1.2 or bivalent Comirnaty (encoding both ancestral strain and BA.4/BA.5 spikes) as the 3^rd^ dose vaccines. The negative control group received 3 doses of formulation buffer. Half of the mice received the 3^rd^ dose on week 29 (26 weeks after 2^nd^ vaccination) and the other half on week 48 (45 weeks after 2^nd^ vaccination). The two cohorts of mice received the 3^rd^ dose of vaccination at different time points because we aimed to assess if the 3^rd^ dose given at a longer interval after the 2^nd^ vaccination would boost the immune response and display efficacy similarly to that given at a shorter interval. At various time points, sera were collected from these mice, and nAb responses against Wuhan-Hu-1 (D614G), Omicron BA.4/BA.5, and Omicron XBB.1.5 pseudoviruses were quantified. As shown in Fig. 3B, two doses of the original monovalent Comirnaty elicited potent nAbs against Wuhan-Hu-1 (D614G), modest levels of nAbs against BA.4/BA.5, and low nAbs against XBB.1.5, at 2 weeks post 2^nd^ vaccination. These nAbs declined during the following-up period until the 3^rd^ vaccination, but not as markedly as seen in human vaccinees who usually exhibit a more notable decrease in nAbs after COVID-19 mRNA vaccinations^45^. The 3^rd^ dose of vaccination increased nAb titers against all 3 pseudoviruses, and the nAb titers raised by M1.2 as the 3^rd^ dose is comparable to the bivalent Comirnaty (*P* > 0.05). Furthermore, the 3^rd^ dose given at both longer and shorter intervals post 2^nd^ vaccination boosted the nAbs comparably to all 3 pseudoviruses, indicating that the bivalent mRNA vaccines as the 3^rd^ dose efficiently recalled memory humoral immune response irrespective of the interval length after the 2^nd^ vaccination.

In Experiment 2, C57BL/6 mice were vaccinated twice, on day 0 and day 21 with 5 µg of the monovalent Comirnaty, followed by receiving 5 µg M1.2 or the bivalent Comirnaty as the 3^rd^ dose vaccine at 11 weeks post 2^nd^ vaccination (Fig. 3A). We chose to give the 3^rd^ dose at this time point because Experiment 1 results showed that after the 2^nd^ vaccination nAbs declined mostly during the first 10 weeks and remained relatively stable thereafter (Fig. 3B). Previous studies also indicated that immune memory reached a relatively steady state at around 3 months after the 2^nd^ dose of SARS-CoV-2 mRNA vaccines^46–48^. Formulation buffer was administered to negative control mice. Three weeks post 3^rd^ vaccination, sera and spleens were harvested from the animals to assess nAb and T cell responses against SARS-CoV-2. Both M1.2 and the bivalent Comirnaty booster dose regimens induced potent nAbs against Wuhan-Hu-1 (D614G), modest levels of nAbs against Omicron BA.1, BA.2, BA.2.12.1, BA.4/BA.5, and barely detectable nAbs against XBB.1.5 (Fig. 3C). The magnitudes of nAbs elicited by M1.2 and the bivalent Comirnaty booster dose regimens were comparable across all tested pseudoviruses. ELISpot results showed that M1.2 as the 3^rd^ dose elicited a potent Th1 biased cytokine T cell response (high IFN-γ spot number and low IL-4 spot number), which is comparable to that induced by the bivalent Comirnaty as the 3^rd^ dose (Fig. 3D and Supplementary Fig. 3A). Furthermore, no significant difference in the spot number of IFN-γ was noted when the T cells were stimulated with either Wuhan-Hu-1 spike peptide pool or XBB.1.5 spike peptide pool, suggesting that the T cells generated in this booster vaccination regimen can equally recognize Wuhan-Hu-1 and XBB.1.5 spike (Fig. 3D). Intracellular cytokine staining (ICS) confirmed the ELISpot results, indicating that M1.2 as the 3^rd^ dose elicited potent Th1-cytokine biased CD8+ and CD4 + T cell responses, which were mostly comparable to those induced by the 3^rd^ dose using the Comirnaty bivalent (Fig. 3E). Generally, CD4+ and CD8 + T cells recognize the Wuhan-Hu-1 and XBB.1.5 spike antigens equally well, as indicated by similar percentage of the cytokine-producing CD4+ or CD8 + T cells after ex vivo stimulation with either of the antigens (Fig. 3E). An exception to this is the IL-2 producing T cells, in which there are more IL-2 producing CD8 + T cells against Wuhan-Hu-1 spike than XBB.1.5 spike from both M1.2 and the Comirnaty bivalent boosted mice. On the contrary, more IL-2-producing CD4 + T cells against XBB.1.5 spike than Wuhan-Hu-1 spike was observed in the Comirnaty bivalent 3^rd^ dose mice. Further experiments are needed to confirm this dichotomy of T cell IL-2 production, its mechanisms, and implications for the vaccine’s efficacy.

Taken together, using a booster vaccination regimen that mimics the human scenario, these results indicate that M1.2 as the 3^rd^ dose induced broad nAb and T cell responses against Wuhan-Hu-1 and Omicron subvariants. Unlike the nAb responses, the magnitudes of T cell responses were overall comparable for Wuhan-Hu-1 and XBB.1.5 spike.

### M1.2 protected mice from multiple Omicron subvariants infection

**Fig. 4:**
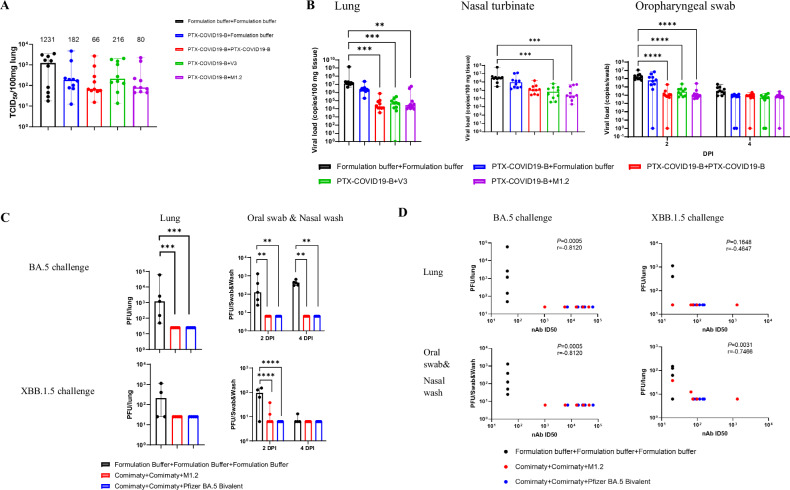
Bivalent mRNA vaccine M1.2 protected mice from Omicron subvariants challenge. **A** Quantity of Omicron BA.1 virus outgrown from lungs of mice. Mice in Fig. 1B were challenged intranasally with 10^5^ TCID_50_ Omicron BA.1 virus. Columns indicate median TCID_50_/100 mg lung tissue, and error bars indicate minimal and maximal values of TCID_50_/100 mg lung tissue. Numbers above the boxes are median values of TCID_50_ per 100 mg lung tissue (*n* = 10). Each dot represents an individual mouse. **B** Quantity of viral genomic RNA copies in tissues from mice in **A**. Columns indicate median viral genomic RNA copy numbers per 100 mg tissue (for lung and nasal turbinate) or per swab sample (for oropharyngeal swabs), and error bars indicate minimal and maximal values of the viral genomic RNA copy numbers (*n* = 10). Each dot represents an individual mouse. **C** Quantity of Omicron BA.5 or XBB.1.5 virus outgrown from lung tissues or Oral swab & Nasal wash samples from mice. Mice in Fig. 3A were challenged intranasally with 10^5^ TCID_50_ Omicron BA.5 virus (*n* = 5) or Omicron XBB.1.5 virus (*n* = 4–5). Columns indicate median PFU per lung or per sample, and error bars indicate minimal and maximal values of the PFU. Each dot represents an individual mouse. **D** Correlation between the quantity of viruses outgrown from mouse samples and nAb ID_50_ titers. Each dot represents one mouse. One-way ANOVA (Kruskal-Wallis test) followed by Dunn’s multiple comparison were used for statistical analysis, except for oropharyngeal swab in **B** and Oral swab & Nasal wash in **C** where two-way ANOVA followed by Tukey’s multiple comparison were used. Spearman correlation was used in the correlation analysis in **D**. *P < 0.05; **P < 0.01; ***P < 0.001; ****P < 0.0001.

To assess the efficacy of M1.2, we first challenged the vaccinated C57BL/6 mice shown in Fig. 1B with 10^5^ TCID_50_ Omicron BA.1 virus at 3 weeks post 2^nd^ vaccination. Previous studies indicated that wild-type C57BL/6 mice were susceptible to BA.1 infection^49,50^. At four days post-infection (DPI), mice sera and lungs were collected. Individual mouse serum was used to measure nAbs against Wuhan-Hu-1 and Omicron subvariants. As shown in Supplementary Fig. 2 and Supplementary Table 1, M1.2 induced potent and broadest nAbs against Wuhan-Hu-1 and Omicron subvariants, confirming the nAb results from the interim sera (1 week post 2^nd^ vaccination) of these mice as shown in Fig. 1C and Table 1. The harvested lungs were used to measure the quantity of BA.1 virus in the lung tissue using a VeroE6-ACE2-TMPRSS2 cell line. Compared with the mice receiving formulation buffer, all vaccine regimens reduced the quantity of BA.1 virus outgrown from the lungs (median TCID_50_/100 mg lung tissue reduced by 82.5–94.6%, Fig. 4A), among which the two doses of PTX-COVID19-B regimen and the PTX-COVID19-B prime-M1.2 boost regimen showed the largest reduction. The decrease in the viral loads in the lungs of PTX-COVID19 vaccinated mice was more prominent in terms of the viral genome copy number measured by real-time RT-PCR, and two doses of PTX-COVID19 mRNA vaccines decreased the viral genome copy number by 2.08 to 2.90 log_10_ compared to the formulation buffer treated group (Fig. 4B). Similar reduction of viral genome copy number in the upper respiratory tract was also noted at 4 DPI in nasal turbinates and 2 DPI in oropharyngeal swabs, in which mice receiving the bivalent M1.2 boost displayed the largest reduction.

We then tested the efficacy of M1.2 as the booster (3^rd^) dose using the K18-hACE2 mice shown in Fig. 3A. Four weeks post 3^rd^ vaccination, the K18-hACE2 mice were challenged with 10^5^ TCID_50_ Omicron BA.5 or XBB.1.5. Mice receiving M1.2 or the bivalent Comirnaty as the 3^rd^ dose were completely protected from Omicron BA.5 challenge, as indicated by no virus outgrowth from either upper respiratory tracts (oral swab & nasal wash samples) or lungs of these mice, while all control mice receiving 3 doses of formulation buffer had abundant outgrown BA.5 virus in these tissues (Fig. 4C). M1.2 or the bivalent Comirnaty also completely protected mice from lung infection by XBB.1.5. No XBB.1.5 virus outgrew from the lung samples collected from the mice receiving either M1.2 or bivalent Comirnaty as the 3^rd^ dose (*n* = 5), while the virus was detected in 2 out of the 4 control mice vaccinated with the formulation buffer. It should be noted that compared to Wuhan-Hu-1 and previous VOCs, Omicron subvariants replicated less well in K18-hACE2 mice^49–51^. The control mice receiving formulation buffer without detectable virus outgrown from their lung tissues might have low level virus replication in their lungs below the limit of detection of our assay. M1.2 and the bivalent Comirnaty also significantly reduced the XBB.1.5 virus quantity in the upper respiratory tract compared to the formulation buffer control (Fig. 4C). The high protection efficacy of the booster vaccination regimen against the XBB.1.5 challenge (Fig. 4C) and the low levels of nAbs against XBB.1.5 in these animals (Fig. 3B) prompted us to do a correlation analysis between nAbs and the quantity of the outgrown virus (Fig. 4D). We found that nAb titers inversely correlated with the quantity of BA.5 virus in both lungs (*P* = 0.0005, r = −0.8120) and upper respiratory tract (*P* = 0.0005, r = −0.8120) samples. However, for the XBB.1.5 challenge, nAb levels were inversely correlated with virus quantity only in the upper respiratory tract (*P* = 0.0031, r = −0.7466) but not in the lungs (*P* = 0.1648, r = −0.4647), suggesting that nAbs may not solely account for the protection against XBB.1.5 infection of lungs in these mice.

### M1.2 protected hamsters from Wuhan-Hu-1 and Omicron BA.2 infection

**Fig. 5:**
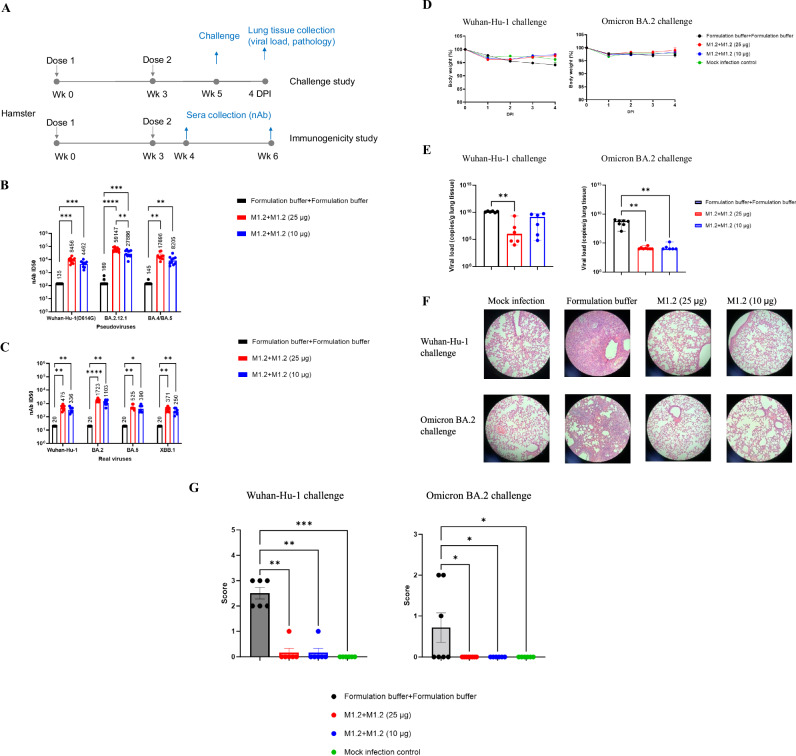
Bivalent mRNA vaccine M1.2 elicit potent and broad nAbs in hamsters and protected the hamsters from Wuhan-Hu-1 and Omicron BA.2 challenge. **A** Schedules for the challenge study and Immunogenicity study in hamsters. Six- to eight-week-old Syrian golden hamsters were vaccinated twice with 10 µg or 25 µg M1.2. In Challenge study, hamsters (*n* = 6–8) were intranasally infected with 10^5^ TCID_50_ of Wuhan-Hu-1 or Omicron BA.2 virus at 2 weeks post 2^nd^ vaccination. In Immunogenicity study, sera (*n* = 10) were collected at 1 week or 3 weeks post 2^nd^ vaccination for nAb analysis. **B** and **C** Sera from hamsters in **A** Immunogenicity study collected at 1 week **B** or 3 weeks **C** post the 2^nd^ vaccination were used in nAb pseudovirus assay **B** or real virus assay **C**. Columns and error bars indicate nAb ID_50_ geometric means±95% confidence interval of individual hamster serum (*n* = 10 for **B**, *n* = 6 or 7 for **C**). Each dot represents an individual hamster. LLOQ of the nAb assay in **B** is 270 and **C** is 40. When serum nAb ID_50_ values were below LLOQ, half of the LLOQ values (135 in **B** and 20 in **C**) were assigned to the sera. The numbers on top of the columns are geometric mean values of the nAb ID_50_. **D** Body weight change after the challenge. Shown are mean percentage of body weight (*n* = 6–8) compared to the baseline body weight (100%, 0 DPI before challenge). Mock infection controls (*n* = 3) were given intranasally cell culture media instead of the viruses. **E** Quantity of viral genomic RNA copies in lungs from hamsters in **A** Challenge study. Columns indicate median viral genomic RNA copy numbers per gram of lung tissue, and error bars indicate minimal and maximal values of the viral genomic RNA copy numbers (*n* = 6–8). Each dot represents an individual hamster. LLOQ for Wuhan-Hu-1 virus genomic RNA copy number is 5129 per gram lung tissue and 9772 for BA.2. **F** Representative pathology pictographs of the lungs collected at 4 DPI. **G** Semiquantitative pathology scores of the lungs collected at 4 DPI. Columns and error bars indicate mean± S.E.M (*n* = 6–8). Each dot represents an individual hamster. Two-way ANOVA followed by Tukey’s multiple comparison was used for statistical analysis in **B** and **C**. One-way ANOVA (Kruskal–Wallis test) followed by Dunn’s multiple comparison was used for statistical analysis in **E** and **G**. *P < 0.05; **P < 0.01; ***P < 0.001; ****P < 0.0001.

Hamster is another widely used animal model for evaluating SARS-CoV-2 vaccines and antiviral drugs^52^. To evaluate the efficacy of M1.2 in protecting hamsters from SARS-CoV-2 infection, Syrian golden hamsters were immunized twice, at an interval of 3 weeks, with 25 µg or 10 µg M1.2 per hamster, and then challenged with the Wuhan-Hu-1 or Omicron BA.2 viruses (inoculum 10^5^ TCID_50_ per animal) at two weeks post the 2^nd^ immunization (Fig. 5A). An immunogenicity satellite group, in which hamsters received immunization of M1.2 at the same dose levels and with the same immunization regimen as the challenge study groups, was included to examine the nAb titers at 1 week or 3 weeks post the second immunization. Similar to the findings from the immunized mice (Fig. 1E), primary series vaccination with M1.2 elicited dosage-dependent potent nAbs in the hamster sera collected at 1 week post the 2^nd^ vaccination, against Wuhan-Hu-1 (D614G), Omicron BA.2.12.1, and BA.4/BA.5 pseudoviruses, with the nAb titers being highest against BA.2.12.1 and lowest against Wuhan-Hu-1 (D614G) (Fig. 5B). The hamster sera collected at 3 weeks post the 2^nd^ vaccination were used in a real virus neutralization assay, and both 25 µg and 10 µg dose group exhibited significant nAbs against ancestral and Omicron viruses, including XBB.1(Fig. 5C).

Consistent with previous reports, upon Wuhan-Hu-1 virus infection, the body weight of the control hamsters receiving formulation buffer decreased from 1-4 DPI (Fig. 5D). M1.2 immunized hamsters showed slight body weight loss on 1 DPI similar to the control animals receiving mock infection, and then gained weight from 2-4 DPI (Fig. 5D). Omicron BA.2 infection did not cause noticeable body weight loss in hamsters receiving formulation buffer compared to the mock infection controls. Geometric mean lung viral load of the 25 µg and 10 µg dose M1.2 immunized hamsters was 4.10 and 2.25 log_10_ lower than the formulation buffer immunized hamsters after Wuhan-Hu-1 virus challenge, and 4.33 and 4.20 log_10_ lower after BA.2 virus infection, respectively (Fig. 5E). Lung histopathology examination showed that after Wuhan-Hu-1 virus infection, all hamsters in the formulation buffer control group showed mild to moderate multifocal mixed cell inflammation, while only 1 out of 6 hamsters immunized with 10 µg M1.2 exhibited very mild focal mixed cell inflammation and only 1 out of 6 hamsters immunized with 25 µg M1.2 displayed very mild focal mononuclear cell infiltration in the adventitia and smooth muscle bundles of bronchi (Fig. 5F,G). After BA.2 virus infection, 3 out of 7 hamsters in the formulation buffer control group showed noticeable pathological changes, with 2 hamsters showing mild multifocal mixed cell inflammation and 1 hamster showing very mild or mild focal mononuclear cell infiltration in the adventitia and smooth muscle bundles of bronchi (Fig. 5F,G). In contrast, the M1.2 immunized hamsters did not exhibit any lung pathological changes after BA.2 virus infection. Taken together, these data demonstrated that vaccination with M1.2 protected hamsters against the SARS-CoV-2 infection by both Wuhan-Hu-1 and Omicron BA.2 viruses.

### Omicron JN.1 subvariant substantially escaped from M1.2 induced nAbs but not T cells

**Fig. 6:**
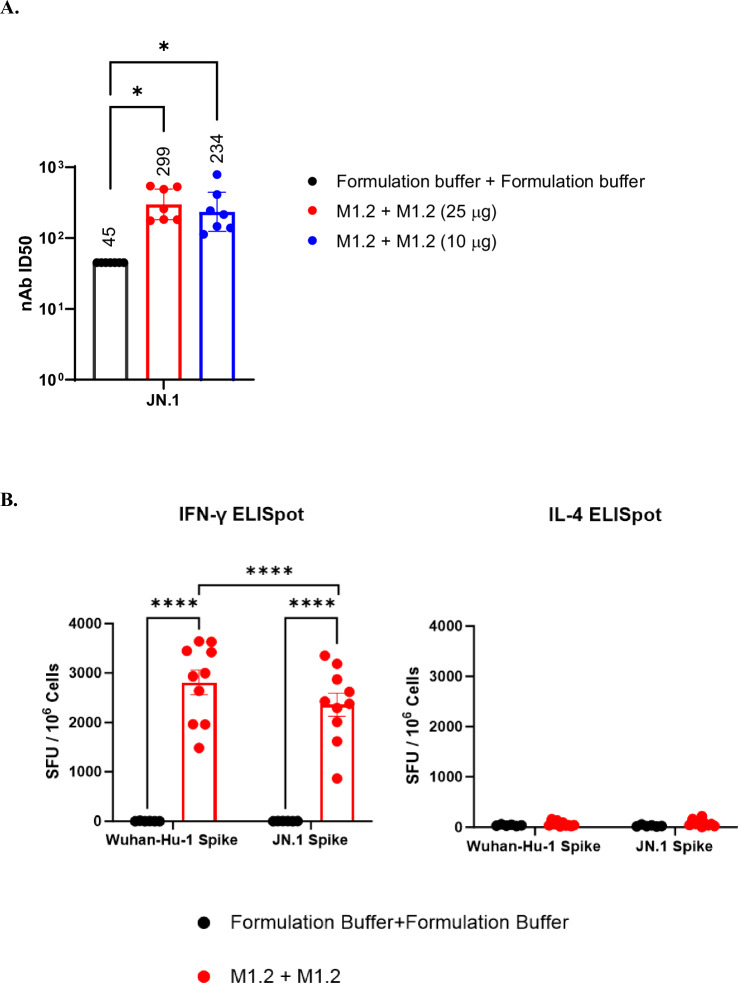
Omicron JN.1 substantially escaped from M1.2 elicited nAbs but not the T cells. **A** Hamster sera used in Fig. 5C were assessed against JN.1 in nAb pseudovirus assay. Columns and error bars indicate nAb ID_50_ geometric means±95% confidence interval of individual hamster serum (*n* = 6 or 7). Each dot represents an individual hamster. LLOQ of the nAb assay is 90. When serum nAb ID_50_ values were below LLOQ, half of the LLOQ values (45) were assigned to the sera. The numbers on top of the columns are geometric mean values of the nAb ID_50_. **B** ELISpot results. Splenocytes from C57BL/6 mice receiving two vaccinations of M1.2 at 10 µg dose (*n* = 10) or control formulation buffer (*n* = 6) were stimulated with Wuhan-Hu-1 spike peptide pool or JN.1 spike peptide pool. Columns and error bars indicate mean± S.E.M. of spot forming units (SFU) per million input cells. Each dot represents an individual mouse. One-way ANOVA (Kruskal–Wallis test) followed by Dunn’s multiple comparison was used for statistical analysis in **A**. Two-way ANOVA followed by Šidák’s multiple comparison was used for statistical analysis in **B**. *P < 0.05; **P < 0.01; ***P < 0.001; ****P < 0.0001.

JN.1 lineage are the currently dominant circulating Omicron subvariants with more mutations than their predecessors and are highly resistant to the neutralization by the sera from subjects who had received BA.5 bivalent mRNA vaccine booster immunization (Supplementary Fig. 4)^53,54^. To evaluate the effect of M1.2 induced nAbs on JN.1 subvariants, hamster sera from the immunogenicity study shown in Fig. 5A were used in neutralization assay against JN.1 pseudovirus. As shown in Fig. 6A, M1.2 vaccinated hamster sera had detectable but low titer nAbs against JN.1. Compared to other Omicron subvariants, JN.1 substantially escaped M1.2 elicited nAbs (Fig. 5B and Fig. 6A). To assess if M1.2 induced T cells can still recognize JN.1, splenocytes from M1.2 vaccinated C57BL/6 mice were stimulated with spike peptide pool from JN.1 or Wuhan-Hu-1 and cytokine production was then measured by ELISpot. As shown in Fig. 6B, M1.2 induced potent Th1-biased T cells response against both Wuhan-Hu-1 and JN.1. Eighty-four percent (84%) of the IFN-γ response against Wuhan-Hu-1 spike remained intact against JN.1 (2813 ± 245 SFU/million cells for Wuhan-Hu-1 spike vs. 2362 ± 234 SFU/million cells for JN.1 spike, mean ± S.E.M.), though the 16% difference was statistically significant. Taken together, these results indicate that JN.1 substantially evades from M1.2 elicited nAb but M1.2 induced T cells can still effectively respond to JN.1.

## Discussion

A key unresolved issue in COVID-19 booster immunization is when the vaccine needs to be updated with the latest circulating VOC’s spike. Many factors need to be considered when making the decision, but the effectiveness of current vaccines against the latest circulating and potential emerging VOCs is a primary determinant^39,40^. Before the emergence of Omicron, booster vaccination with the prototype COVID-19 mRNA vaccines showed high VE in protection against symptomatic COVID-19 infections caused by pre-Omicron VOCs^55^. Extensive mutation of Omicron spike enabled the virus to escape from the nAb response induced by this booster immunization accompanied by remarkable VE reduction, which necessitated their update to bivalent mRNA vaccines that incorporated the then-dominant Omicron BA.4/BA.5 spike^14,15,56^. Observational studies of human vaccinees revealed that these bivalent mRNA vaccines remained effective, especially for severe disease and death, against co-circulating Omicron subvariants other than BA.4/BA.5, including Omicron XBB subvariants, even though XBB subvariants are highly resistant to the nAbs induced by the bivalent mRNA vaccines^57–60^. By using mice and hamster models, results reported here indicate that our bivalent spike-based mRNA vaccine, M1.2, used as primary series or booster vaccination can provide a broad protection against upper and lower respiratory tract infection by Wuhan-Hu-1 and Omicron subvariants, especially the subvariants antigenically distant from the Omicron BA.2.12.1 spike protein encoded in M1.2, such as BA.1, BA.5 and XBB.1.5 (see Fig. 1A and antigenic cartography in refs. ^61,62^ for antigenic distances between BA.2.12.1 and Omicron subvariants). Our findings are consistent with the human observational study data and suggest a bivalent mRNA vaccine probably does not need to match circulating or emerging VOCs to be effective. Whether this will also hold true for a monovalent updated mRNA vaccine such as the ones recommended for 2023 fall season requires close monitoring of human VE data and further animal studies.

nAb titers are often used to predict VE and recently were used as a criterion in the decision to update COVID-19 vaccines^39,40^. In this study, we found that M1.2 elicited potent and broad nAb responses against Wuhan-Hu-1 and Omicron subvariants except XBB.1.5, for which only low to modest level of nAbs were detected even after a booster dose (Figs. 1E, 3B, C). This corroborates the high immune evasiveness of XBB.1.5 reported in literature^17,20,63–67^. However, the 3-dose vaccination regimen used here still defended K18-hACE2 mice against the XBB.1.5 challenge and the efficacy of protection against the XBB.1.5 lung infection did not correlate with the anti-XBB.1.5 nAb titers (Fig. 4D). It is possible that the low to modest level of nAbs could still protect the lungs from XBB.1.5 infection, or non-nAbs mechanisms, such as non-neutralizing Abs and T cells^20,24,25^, mediated the immune protection of the lungs. In contrast, anti-XBB.1.5 nAb titers correlates with the vaccine’s efficacy in protection against upper respiratory tract infection, as shown by the inverse correlation between the nAb titer and virus quantity in the nasal swab and oral wash samples. Our results are consistent with the reported discrepancy between nAbs and COVID-19 vaccine VE in protection against death and severe disease^68^. Although current COVID-19 mRNA vaccines administered by intramuscular route are poor in inducing mucosal immune responses^69–71^, they can protect animals from SARS-CoV-2 infection of the upper respiratory tract, possibly due to the neutralizing IgG exudating from systemic circulation^22,42,70,71^. Thus, nAbs may be more important in preventing SARS-CoV-2 infection of the upper respiratory tract than that of the lower respiratory tract. Mucosal mRNA vaccines are under development, and it will be interesting to investigate if these mucosal mRNA vaccines can provide a broad protection of both the upper and lower respiratory tracts against SARS-CoV-2 VOCs infection. Furthermore, consistent with previous reports, our findings indicate that nAb levels may not always be a reliable surrogate for COVID-19 vaccine’s VE in protection against lung infection and severe disease and needs to be cautiously used as evidence in the decision-making process to update COVID-19 vaccines.

We found that both the primary series (Fig. 2) and a booster dose (Fig. 3D,E) of M1.2 induced broad and potent T cell responses against Wuhan-Hu-1 and Omicron subvariants. Indeed, the cytokine production profiles of the T cells from the mice receiving M1.2 vaccine are largely similar when stimulated ex vivo with spike peptide pools from Wuhan-Hu-1 or Omicron BA.4/BA.5 or XBB.1.5. This is consistent with previous reports, demonstrating that T cell responses elicited by primary series of prototype mRNA vaccines and booster mRNA vaccines remain largely intact against Omicron subvariants^30,32–34,63,72–75^. In contrast, we found that T cells elicited by primary series vaccination with Omicron spike-based monovalent mRNA vaccine were more reactive to Omicron spike than Wuhan-Hu-1 spike (Fig. 2B). Furthermore, nAbs induced by Omicron spike-based monovalent mRNA vaccine was also potent in strength but narrower in breadth than bivalent mRNA vaccine (Fig. 1C,E, Table 1, Supplementary Fig. 2, and Supplementary Table 1). Others have previously reported that a primary series of Omicron spike-based monovalent mRNA vaccines elicited a narrow nAb response against the homologous Omicron subvariant, but little was reported on the breadth of the T cell response in these studies^18,76^. Together with these previous reports, our findings raise a concern that primary series vaccination using Omicron spike monovalent vaccine may elicit a potent but narrow immune response. Given that a monovalent XBB.1.5 spike mRNA vaccine is currently recommended as a primary series vaccination for 6 month to 4-year-old infants and children^77^, further studies are needed to examine the breadth of immune response upon primary series vaccination using monovalent Omicron spike mRNA vaccine and its effect on VE.

Consistent with previous reports^19,20,37,38,65^, our results indicated that booster vaccination with M1.2 or Comirnaty bivalent vaccine after the primary series immunization with prototype mRNA vaccine preferentially enhance the nAb response against ancestral spike encoded by the primary series vaccine rather than generate nAbs against Omicron spike encoded in the 3^rd^ dose bivalent vaccine (Figs. 3B and 3C), confirming the presence of immune imprinting on nAb response induced by COVID-19 mRNA vaccines^16,20^. In contrast, we found that T cells after bivalent mRNA vaccine booster dose respond almost equally well to both ancestral and Omicron spike antigens (Fig. 3D,E). Compared to nAb response, less is known about immune imprinting on T cells^41,78^. Most reports showed that T cells elicited by COVID-19 vaccines were not impaired in recognizing VOC spike proteins^30,32–34,63,72–75^. Our findings on T cell response in the mice receiving booster doses are consistent with these reports, indicating little possibility of immune imprinting on T cells induced by the vaccination regimen using prototypical vaccines as primary series and bivalent vaccines as the booster dose. On the other hand, a few studies reported VOCs spike can escape from T cells elicited by previous infection or vaccination^79–81^ and one of these studies suggested that immune imprinting may suppress the generation of T cells specific for the Omicron spike^79^. In this regard, as mentioned above, we noticed that primary series vaccination with monovalent Omicron spike elicited T cells that respond better to Omicron spike than to Wuhan-Hu-1 spike (Fig. 2B). Thus, further studies in animal models and humans are needed to examine if the T cell response will be immune imprinted by monovalent Omicron spike vaccine as primary series or booster vaccinations.

Two mechanisms may account for the breadth of M1.2 elicited T cell responses against Omicron subvariants. First, the mutations in the spikes of the Omicron subvariants mainly occur in the B cell epitopes not the T cell epitopes. Indeed, only 1 of 8 wild-type spike T cell epitopes was mutated in Omicron BA.1 for either BALB/c or C57BL/6 mice^32,82^. Majority of T cell epitopes are also conserved in Omicron subvariants, including JN.1, in humans^32,83^. Second, M1.2 elicited high frequencies of T cells can still efficiently respond to the mutated T cell epitopes. Previous studies support this mechanism^32–34^. Additional studies, such as using T cell clones from M1.2 vaccinated mice to compare their reactivity against wild type and mutated epitopes, are needed to elucidate the mechanisms underlying the breadth of M1.2 induced T cell responses.

Our study has some limitations. First, we used mice and hamster models. Although these models were widely used in evaluating COVID-19 vaccine’s efficacy, they cannot completely replicate humans, for example, infants or individuals who are immunocompromised or have other comorbidities. Therefore, our results must be interpreted cautiously to translate into human scenarios. Second, we only examined nAb and T cell responses and did not evaluate the contribution of other immune mechanisms, such as antibody-dependent cell-mediated cytotoxicity, to the vaccine’s efficacy. Third, T cell responses were analyzed in cohorts of mice different from the challenged mice. We could not check T cell responses in challenged mice due to biosafety concerns and thus cannot perform correlation analysis of T cell response with the vaccine’s efficacy in the same challenged animals. We did not deplete nor adoptively transfer T cells to animals to directly demonstrate T cell’s role in the vaccine’s efficacy. Fourth, the animals were challenged at 2-4 weeks post 2^nd^ or 3^rd^ vaccination, and thus our results may only apply to the exposure to SARS-CoV-2 happening shortly after vaccination when the immune response has not declined from the peak. However, M1.2 vaccinated mice with low to undetectable levels of pre-challenge nAbs against XBB.1.5 were all protected from XBB.1.5 infection and spike-specific T cells were likely to mediate the protection (Fig. 3D,E, Fig. 4C,D). Spike-specific T cells are more durable than nAbs and could maintain at a relatively stable level up to 6 months or longer after the vaccination^84,85^. Therefore, M1.2 booster vaccination may provide protection against symptomatic or severe Omicron subvariant infections for half a year or even longer. Long term VE of mRNA vaccines against symptomatic or severe infections caused by emerging Omicron subvariants is not clear yet, and more animal and clinical studies are needed to answer this important question. Fifth, we only studied mRNA vaccines, and thus our results may not apply to other COVID-19 vaccine platforms. All these limitations need to be addressed in future studies.

In summary, we found our bivalent mRNA vaccine M1.2 elicited potent and broad immune responses in small animal models and exhibited high efficacy in protecting these animals from Omicron subvariants’ challenges. T cells were likely to play an important role in mediating the protection, especially for antigenically distant Omicron subvariants. Our findings suggest that current bivalent mRNA vaccines (containing the initial Omicron spike variants) may still be able to protect vaccinees from circulating and emerging VOCs’ infections, especially from severe disease, and support a COVID-19 immunization strategy distinct from current influenza vaccine’s annual update approach^86^. Furthermore, our findings highlight the urgency to develop a T cell vaccine incorporating more conserved SARS-CoV-2 proteins other than spike in a vaccine formulation to provide broad protection against emerging VOCs, which will benefit low- and middle-income countries that cannot afford frequent updates of the vaccines.

## Methods

### Ethics

All animal work was approved by the Animal Care Committees of The University of Toronto, University of Calgary, and Wuhan Institute of Virology.

### Vaccine

Our prototypical mRNA vaccine, PTX-COVID19-B, encodes the full-length spike from SARS-CoV-2 Wuhan-Hu-1 isolate, GenBank accession number: MN908947.3, with a D614G substitution^42,43^. Monovalent mRNA vaccine V3 encodes the full-length spike from SARS-CoV-2 BA.2.12.1, and bivalent mRNA vaccine M1.2 is the 1:1 mass ratio mixture of PTX-COVID19-B and V3. The mRNAs in these vaccines contain codon-optimized open reading frames for the spike proteins flanked by an optimized capped 5’ UTR and an optimized 3’ UTR followed by a poly-A tail. Production, purification, and characterization methods for these mRNA vaccines have been published before^42^. Briefly, mRNA in vitro transcribed by T7 RNA polymerase was encapsulated in LNPs composed of 4 lipids (DSPC, cholesterol, PEG-lipid and ionizable lipid) (Vancouver, BC, Canada), concentrated by tangential flow ultrafiltration, diafiltered against an aqueous buffer system, and sterilized through 0.2 µm filter. All mRNA vaccines used in this study passed the quality tests at Providence Therapeutics: mRNA encapsulation efficiency was≥ 94%, PDI was ≤ 0.20, particle sizes were 71 nm to 76 nm, mRNA sizes were as expected from the full-length spike plus 5’UTR and 3’ UTR, mRNA integrity was ≥ 85%, and capping efficiency was ≥ 85%.

### Mouse and Hamster vaccination

Female C57BL/6 mice (Charles River Canada, Saint-Constant, QC, Canada) or K18-hACE2 mice (B6.Cg-Tg(K18-ACE2)2Prlmn/J, JAX strain # 034860, Jackson Lab, Bar Harbor, ME) of 6- to 8-week-old were vaccinated intramuscularly twice or 3 times with a time interval as specified in Figs.1B, 1D, and 3A. 5 µg or 10 µg mRNA vaccines in 50 μl total volume were injected into the hind leg muscle for each immunization. Naïve mice received the same volume of vaccine formulation buffer. At various time points post vaccinations, blood was collected from the mice. For some experiments, spleens were also collected from humanely euthanized mice at the end of the study. Serum was isolated from the blood by centrifugation at 10,000 g for 30 s at 4 °C at University of Toronto for C57BL/6 mice, or 3,000 *g* for 10 min at University of Calgary for K18-hACE2 mice.

Six- to eight-week-old male Syrian golden hamsters (Charles River) were vaccinated twice with a 3-week interval. Ten µg or 25 µg M1.2 (22 hamsters received 10 µg dose, and 24 hamsters received 25 µg dose) were injected via intramuscular route into rear limbs. Placebo control group (29 hamsters) received the vaccine formulation buffer. The immunized hamsters in each group were divided into a satellite subgroup (10 hamsters) and a challenge study subgroup (the remaining hamsters). One week and 3 weeks after the second vaccination, blood samples were collected from hamsters in the satellite subgroups for neutralizing antibody tests, using pseudoviruses or real viruses, respectively. The serum was isolated from the blood by centrifugation at 10,000 *g* for 30 s at 4 °C before subsequent tests.

### Serum neutralization using pseudovirus

nAbs in animal’s sera were detected using spike-pseudotyped lentiviral assays as described before^42,43^. Briefly, diluted mouse sera (1:80 from stock sera except 1:40 for XBB.1.5 virus) and hamster sera (1:270 as the starting dilution) were serially diluted and incubated with SARS-CoV-2 pseudoviruses for 1 h at 37 °C before being added to HEK293T-ACE2/TMPRSS2 cells (for mouse sera) or Vero cells (for hamster sera). The cells were then incubated for an additional 48 h at 37 °C, lysed, and Bright-Glo luciferase reagent (Promega, Madison, WI) was added for 2 minutes before reading with a PerkinElmer Envision instrument (PerkinElmer, Waltham, MA). The 50% neutralization titer (ID_50_) were calculated with nonlinear regression (log[inhibitor] versus normalized response – variable slope) using GraphPad Prism 9.5 (GraphPad Software, La Jolla, CA). Lower limit of quantitation (LLOQ) was defined as the starting dilution of the sera (for mouse 1:80 except 1:40 when testing against XBB.1.5 pseudovirus; for hamster 1:270 except 1:90 when testing against JN.1 pseudovirus). nAb ID_50_ values calculated lower than LLOQ were assigned ½ of the LLOQ (for mouse 1:40 except 1:20 when testing against XBB.1.5 pseudovirus; for hamster 1:135 except 1:45 when testing against JN.1pseudovirus).

### Serum neutralization using real SARS-CoV-2 virus

Serial dilutions of hamster sera were mixed with equal volume of 100 50% cell culture infectious dose (CCID_50_) live viruses. The mixture was incubated at 37 °C for 2 h before adding into Vero cells seeded on 96-well plate, and the cytopathic effects were recorded 4 days later. The Spearman-Karber method was used to calculate the nAb titer ID50 (the highest dilution factor of a serum which provided 50% protection). LLOQ was defined as the starting dilution of the sera (1:40). nAb ID50 values calculated lower than LLOQ were assigned ½ of the LLOQ (1:20).

### ELISpot assay

ELISpot was done using mouse IFN-γ and IL-4 ELISpot kit (Mabtech, Cincinnati, OH) according to the manufacturer’s protocol. Briefly, ELISpot plates pre-coated with anti-mouse IFN-γ or anti-mouse IL-4 antibody were washed and blocked with RPMI-10 medium (RPMI-1640 supplemented with 10% FBS, 100U penicillin, 100 μg streptomycin, and 2mM L-glutamine. All were purchased from Wisent Bioproducts, St-Bruno, QC, Canada) for at least 30 min. Two hundred fifty thousand (250,000) splenocytes were added into the plates and stimulated with a SARS-CoV-2 spike peptide pools (15-mer peptides with 11 amino acids overlap covering the full-length spike of Wuhan-Hu-1, Omicron BA.4/BA.5, XBB.1.5, or JN.1, JPT Peptide Technologies GmbH, Berlin, Germany) at 1 μg/ml/peptide. Negative control wells were treated with same volume of 40% DMSO (Sigma-Aldrich, Oakville, ON, Canada), which was used to dissolve the peptide pools. PMA/Ionomycin (Sigma-Aldrich) or ConA (Sigma-Aldrich) were used as the positive control. The number of the spike-specific spots was calculated by subtracting the number of the spots of the DMSO control wells from the number of the spots of the corresponding spike peptide pool stimulation wells.

### T cell intracellular cytokine staining (ICS)

T cell ICS was reported previously^42^. Mouse splenocytes were stimulated with the spike peptide pools at 1 μg/ml/peptide in the presence of GolgiStop™ and GolgiPlug™ (BD Biosciences, Mississauga, ON, Canada) for 6 hours. Negative and positive control was treated with 40% DMSO and PMA/Ionomycin (Sigma-Aldrich), respectively. Cells were stained with LIVE/DEAD™ Fixable Stain (Thermo Fisher Scientific, Mississauga, ON, Canada) and a panel of fluorochrome-labelled antibodies including anti-mouse CD3/CD4/CD8/CD44/CD62L/ IFN-γ/TNF-α/IL-2/IL-4/IL-5 mAbs (all purchased from Biolegend, San Diego, CA except CD44 from BD Biosciences). FlowJo (BD Biosciences) was used to analyze the data. For data shown in Fig. 2B, 68,000 (minimum)–190,000 (maximum) splenocyte events were collected, and for Fig. 3E, 50,000 (minimum)-100,000 (maximum) splenocyte events were collected, for each of the samples. The percentage of cytokine^+^ T cells was calculated by subtracting the percentage of the DMSO control cells from the percentage of the corresponding spike peptide pool stimulation cells.

### Mouse SARS-CoV-2 challenge

Challenge viruses Omicron BA.1, BA.5, and XBB.1.5 were obtained from ATCC/BEI Resources and amplified with VeroE6-ACE2-TMPRSS2 cells. Mice were anesthetized by isoflurane inhalation and then intranasally challenged with 10^5^ TCID_50_ SARS-CoV-2 as described before^42^. Body weight and clinical signs were checked before and after the challenge. Oropharyngeal swabs and nasal washes were collected on 2 and 4 DPI. On 4 DPI, mice were humanely euthanized by isoflurane overdose (5% isoflurane gas at a flow rate of 1 L of oxygen per minute) followed by cardiac puncture and exsanguination. Blood and lungs were collected for further analysis.

### Hamster SARS-CoV-2 challenge

Hamsters were challenged using the method reported before^42^. Briefly, hamsters were anesthetized by i.p. administration of 200 mg/kg Avertin before intranasally infected with 10^5^ TCID_50_ SARS-CoV-2 Wuhan-Hu-1 or Omicron BA.2. Mock infection hamsters, which were mock immunized with formulation buffer, were given cell culture medium intranasally. After virus inoculation, animals were monitored daily for body weight and signs of disease or distress. At 4 DPI, animals were humanely euthanized by 3–4% isoflurane inhalation with 0.41 mL/min at 4 L/min fresh gas flow. Lungs were collected for viral load measurement and pathology examination.

### Determination of infectious SARS-CoV-2 titer

Infectious SARS-CoV-2 titer in the lungs of BA.1 challenged C57BL/6 mice was measured at University of Toronto using the reported method^42^ with modification of using VeroE6-ACE2-TMPRSS2 cells instead of VeroE6 cells, and the quantity of the outgrown virus was expressed as TCID_50_, which is the highest dilution factor of the inoculum that yielded 50% of the cells with CPE.

Infectious SARS-CoV-2 titer in the lungs and oral swabs and nasal washes of BA.5 or XBB.1.5 challenged K18/hACE2 mice was measured at University of Calgary according to a previously published protocol^87^. Briefly, confluent monolayers of Vero E6 cells expressing human TMPRSS2 (Vero-TMPRSS2) in 12-well plates were infected with serial tenfold dilutions of each sample (dilutions from 10 to 1,000 for oronasal washes; dilutions from 10 to 10^6^ for lung tissues homogenates). After 1 h incubation, cells were covered in a colloidal cellulose overlay and cultured for 72 h, after which the cells were fixed and stained with crystal violet solution to reveal viral plaques. Plaques were counted manually, and titers were calculated from an averaging of duplicate wells. An aliquot of the virus used to infect the mice, the original inoculum, served as the positive control for plaque formation; virus-free infection medium served as the negative control. The limits of detection (LOD) were 12.5 plaque-forming units (PFU) per sample for the oronasal washes and 50 PFU per lung. Samples with PFU lower than the LOD were assigned with a value of ½ of the LOD value (6.25 PFU for oronasal sample and 25 PFU for lung sample).

### Real-time RT-PCR

Real-time RT-PCR to quantify the genomic copies of SARS-CoV-2 in mouse tissue homogenates was done as described before^42^. Briefly, Luna Universal Probe One-step RT-qPCR kit (New England Biolabs, Ipswich, MA) was used to amplify the envelope (E) gene and an E gene DNA standard (pUC57-2019-nCoV-PC:E, GenScript, Piscataway, NJ) was run at the same time to convert Ct values to genomic copies, by using the Rotor-Gene Q software (QIAGEN). To quantify viral loads in hamster lung tissues, HiScript II One Step qRT-PCR SYBR Green kit (Vazyme Biotech) was used to amplify spike gene with the following primers: RBD-qF1, 5’-CAATGGTTAAGGCAGG-3’; RBD-qR1: 5’-CTCAAGGTCTGGATCACG-3’. The One Step SYBR Green qRT-PCR cycling parameters were as follows: reverse transcription at 50 °C for 3 min, initial denaturing at 95 °C for 30 s, and followed by 40 cycles of amplification (95 °C for 10 s and 60 °C for 30 s).

### Pathology

Lung pathology was examined as reported before^42^. The formalin-fixed hamster lung tissue was embedded in paraffin, sectioned, and then stained with hematoxylin and eosin. Histological sections were examined by certificated pathologists blind to the vaccination status. Lung pathology was graded semi-quantitatively according to Table S1 in our previous report^42^.

### Statistical analysis

One-way ANOVA (Kruskal-Wallis test) followed by Dunn’s multiple comparison and two-way ANOVA followed by Šidák’s or Tukey’s multiple comparison were used for comparison between groups, as indicated in the figure legends. The Spearman correlation test was used for correlation analysis. All statistical analysis was performed using GraphPad Prism 10 (GraphPad Software). *P* < 0.05 was regarded as statistically significant.

## Supplementary information

Supplementary Information

## Data Availability

The datasets used and/or analyzed during the current study are available from the corresponding authors on reasonable request.
