# Supplementary material for: A bivalent COVID-19 mRNA vaccine elicited broad immune responses and protection against Omicron subvariants infection
Source: NPJ Vaccines. 2025 Jan 10;10:4. doi: 10.1038/s41541-025-01062-8 (PMC11718203; doi:10.1038/s41541-025-01062-8)
Supplement: Supplementary file 1 — Supplementary Information [file 41541_2025_1062_MOESM1_ESM.pdf]

Supplementary Figure 1

A.

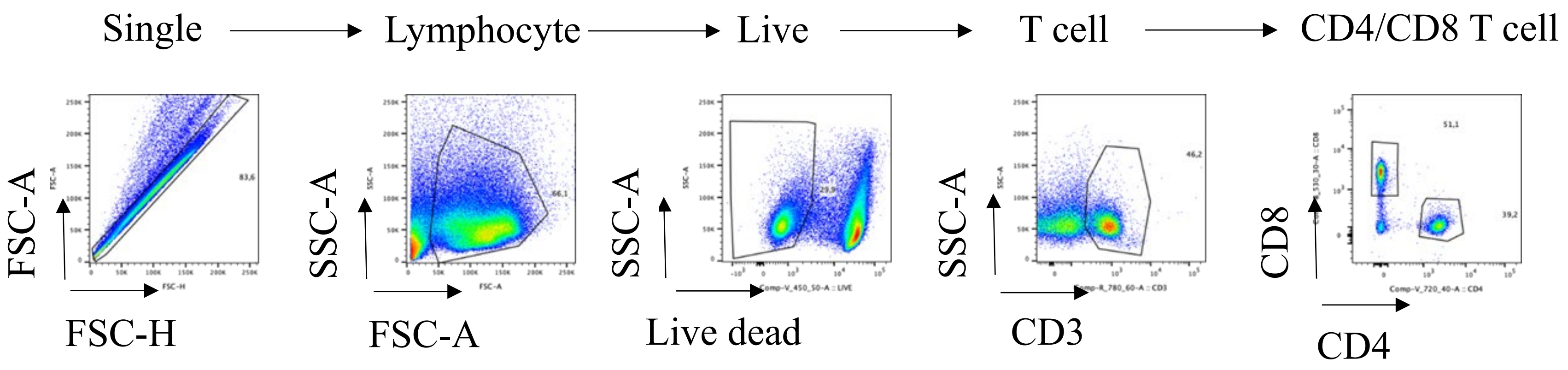

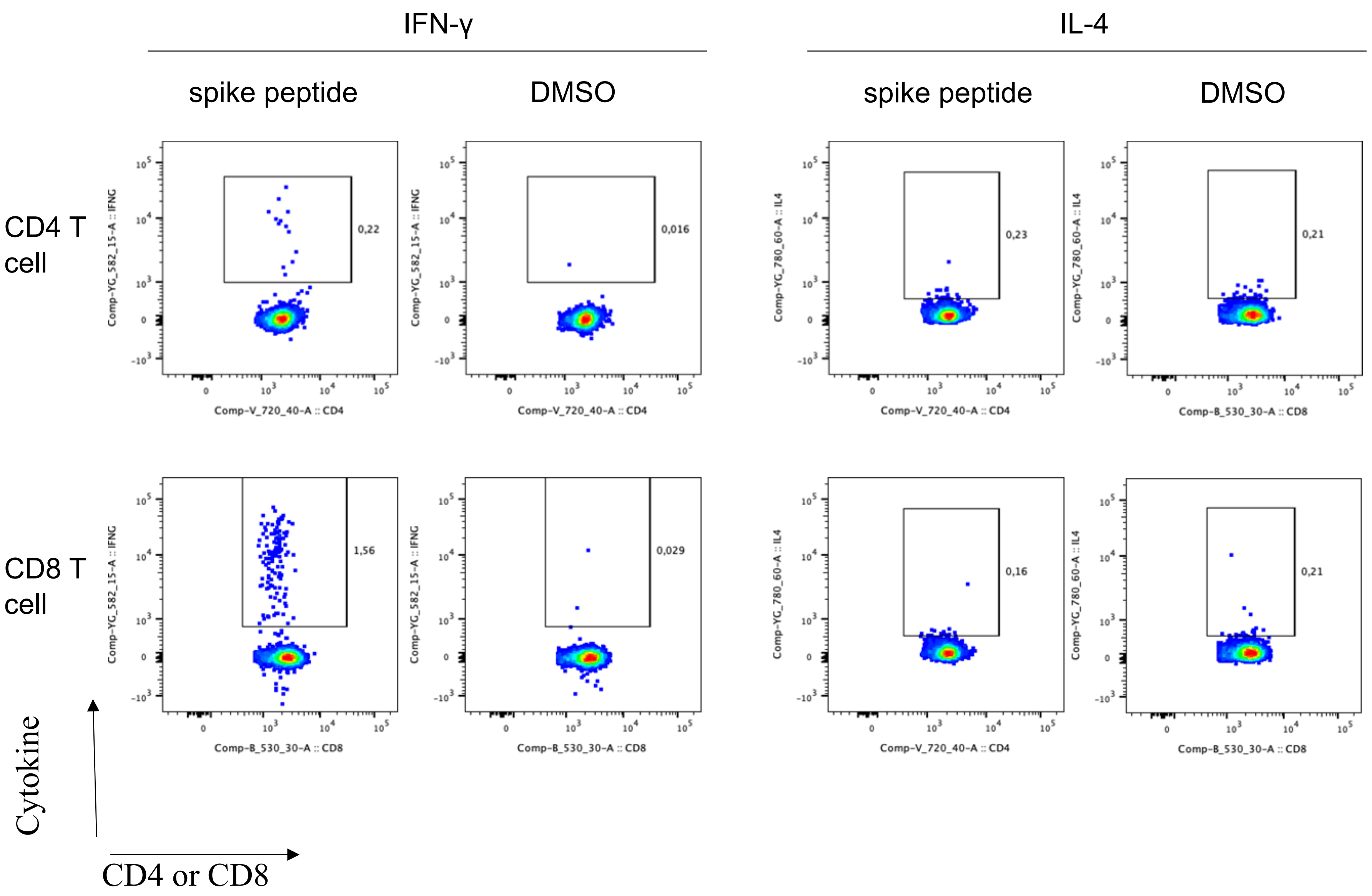

IFN-γ
IL-4
spike peptide
DMSO
spike peptide
DMSO
CD4 T cell
CD8 T cell
0,22
0,016
0,23
0,21
1,56
0,029
0,16
0,21
Comp-YG_582_15-A :: IFNG
Comp-YG_780_60-A :: IL4
Comp-V_720_40-A :: CD4
Comp-B_530_30-A :: CD8
Cytokine
CD4 or CD8

B.

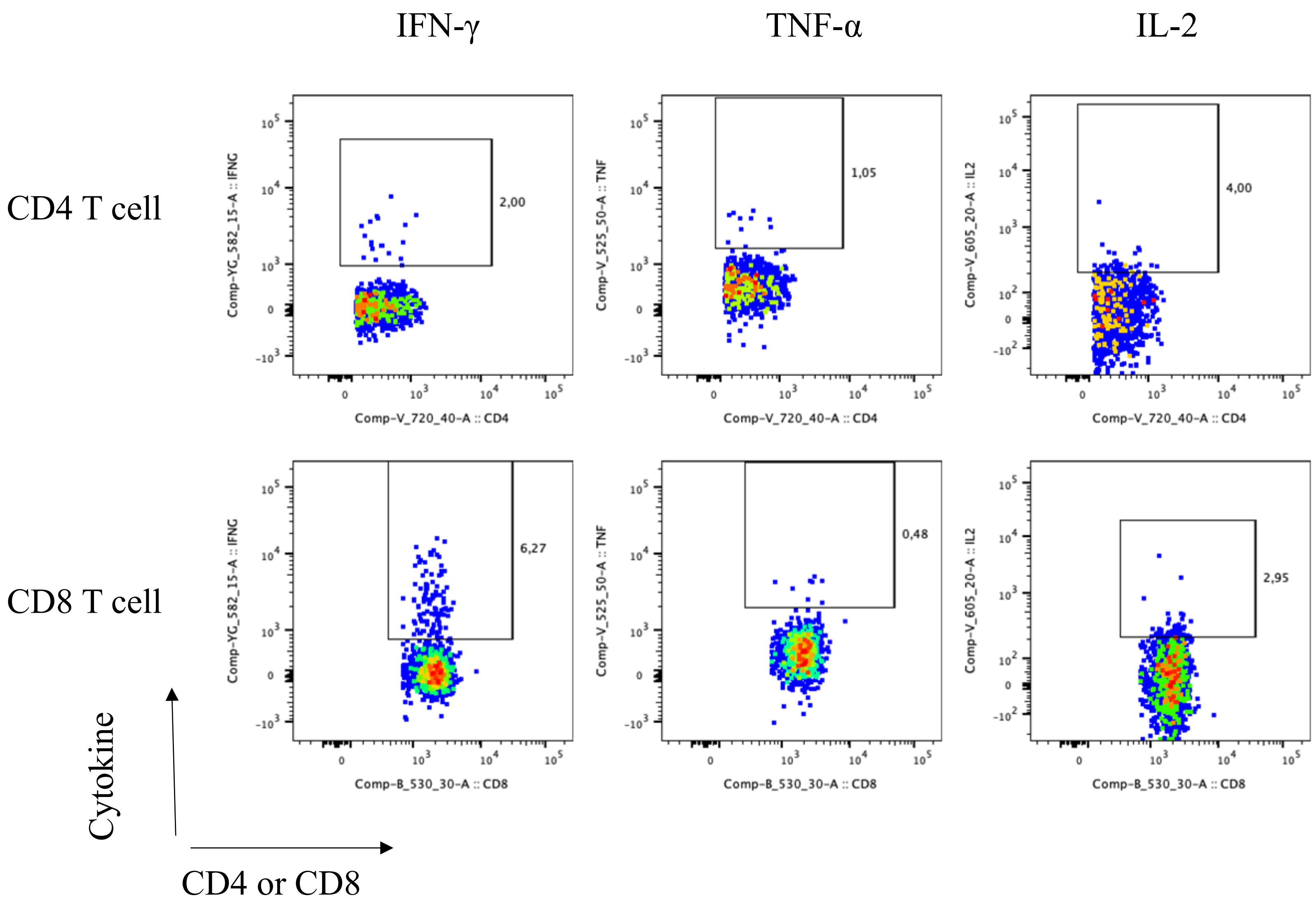
IFN-γ
TNF-α
IL-2
CD4 T cell
CD8 T cell
Comp-YC_582_15-A :: IFNG
Comp-V_525_50-A :: TNF
Comp-V_605_20-A :: IL2
Comp-V_720_40-A :: CD4
Comp-B_530_30-A :: CD8
2,00
1,05
4,00
6,27
0,48
2,95
Cytokine
CD4 or CD8

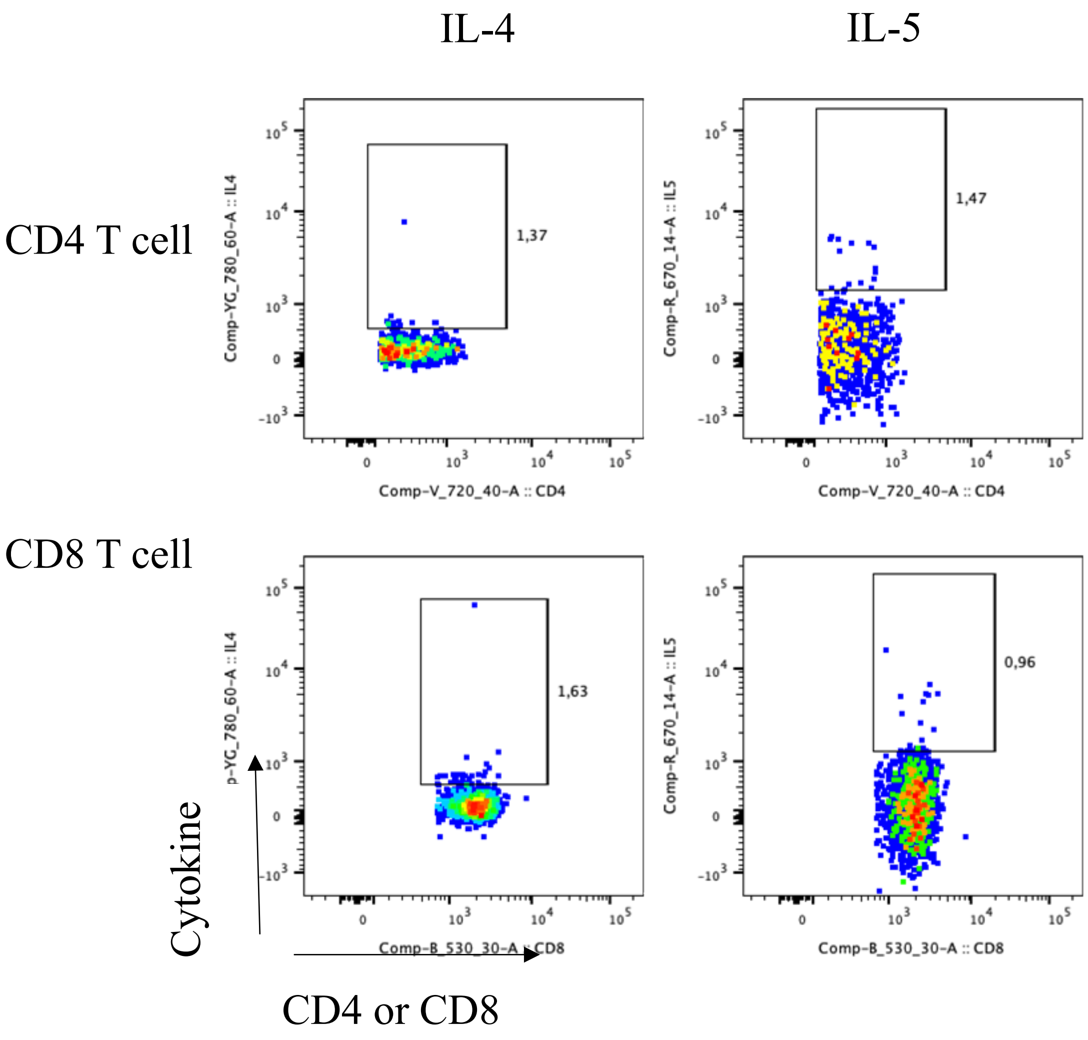
IL-4
IL-5
CD4 T cell
CD8 T cell
1,37
1,47
1,63
0,96
Comp-YC_780_60-A :: IL4
Comp-R_670_14-A :: IL5
Comp-V_720_40-A :: CD4
p-YC_780_60-A :: IL4
Comp-B_530_30-A :: CD8
Cytokine
CD4 or CD8

C.

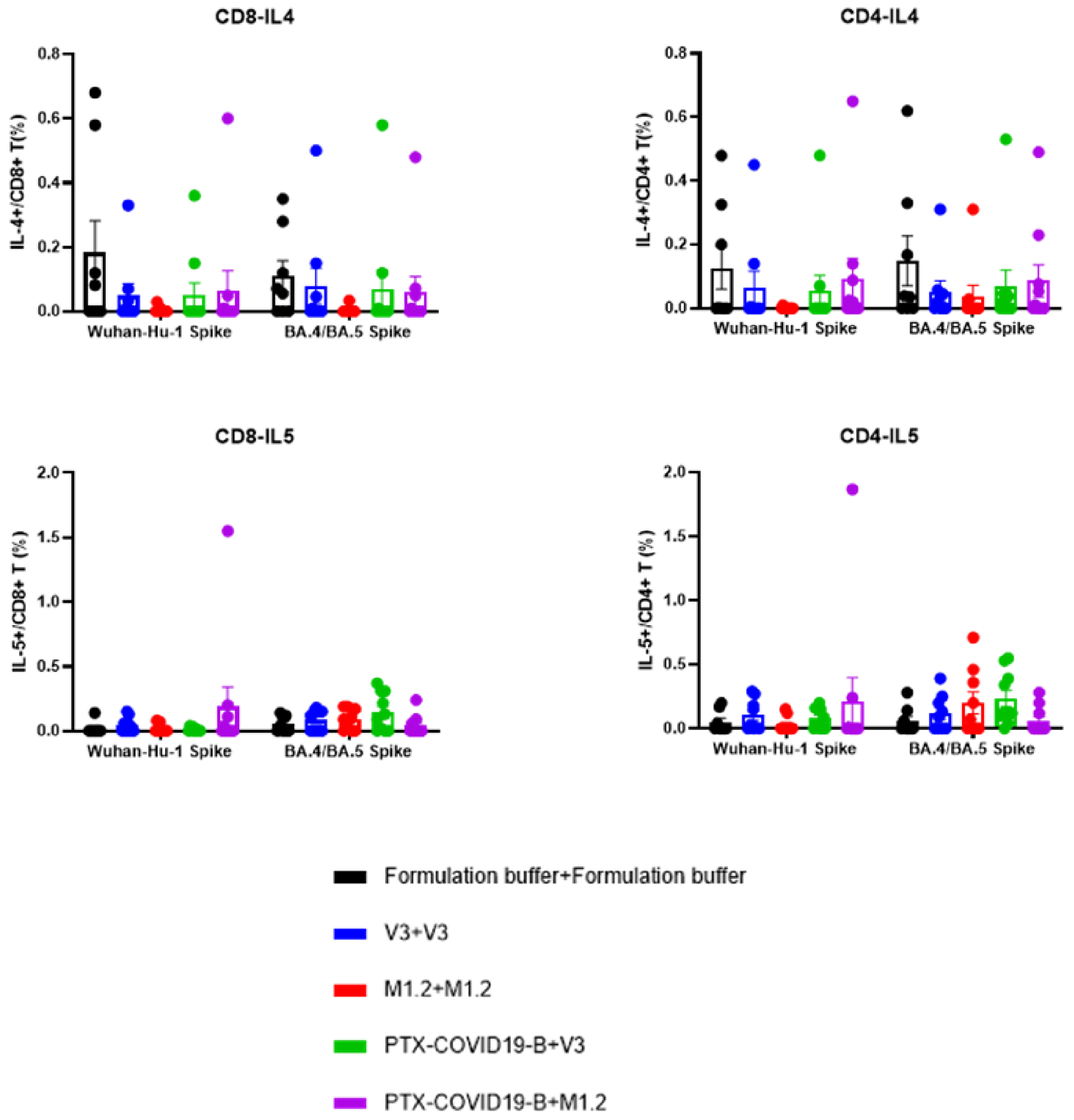
CD8-IL4
IL-4+/CD8+ T(%)
0.8
0.6
0.4
0.2
0.0
Wuhan-Hu-1 Spike
BA.4/BA.5 Spike
CD4-IL4
IL-4+/CD4+ T(%)
CD8-IL5
IL-5+/CD8+ T (%)
2.0
1.5
1.0
0.5
0.0
CD4-IL5
IL-5+/CD4+ T (%)
Formulation buffer+Formulation buffer
V3+V3
M1.2+M1.2
PTX-COVID19-B+V3
PTX-COVID19-B+M1.2

Intracellular cytokine staining of T cells from the mice shown in Fig. 2 (B). (A) Gating strategy used in flow cytometry analysis. IFN-γ and IL-4 graphs from one mouse are shown as an example of the cytokine productions from CD4+ and CD8+ T cells after stimulation with Wuhan-Hu-1 spike peptide pool or DMSO control. **(B)** Representative graph of Th1 and Th2 cytokine production from splenocytes treated with PMA and Ionomycin. **(C)** IL-4 and IL-5 cytokine production of the T cells**.** Mouse splenocytes were stimulated with peptide pools from Wuhan-Hu-1 or Omicron BA.4/BA.5 spike proteins. Column and error bars indicate mean± S.E.M. of percentage of cytokine positive cells in total CD4+ or CD8+ T cells (n=8-10). Each dot represents an individual mouse. Two-way ANOVA followed by Tukey's multiple comparison was used for statistical analysis. No statistical significance was observed.

Supplementary Figure 2

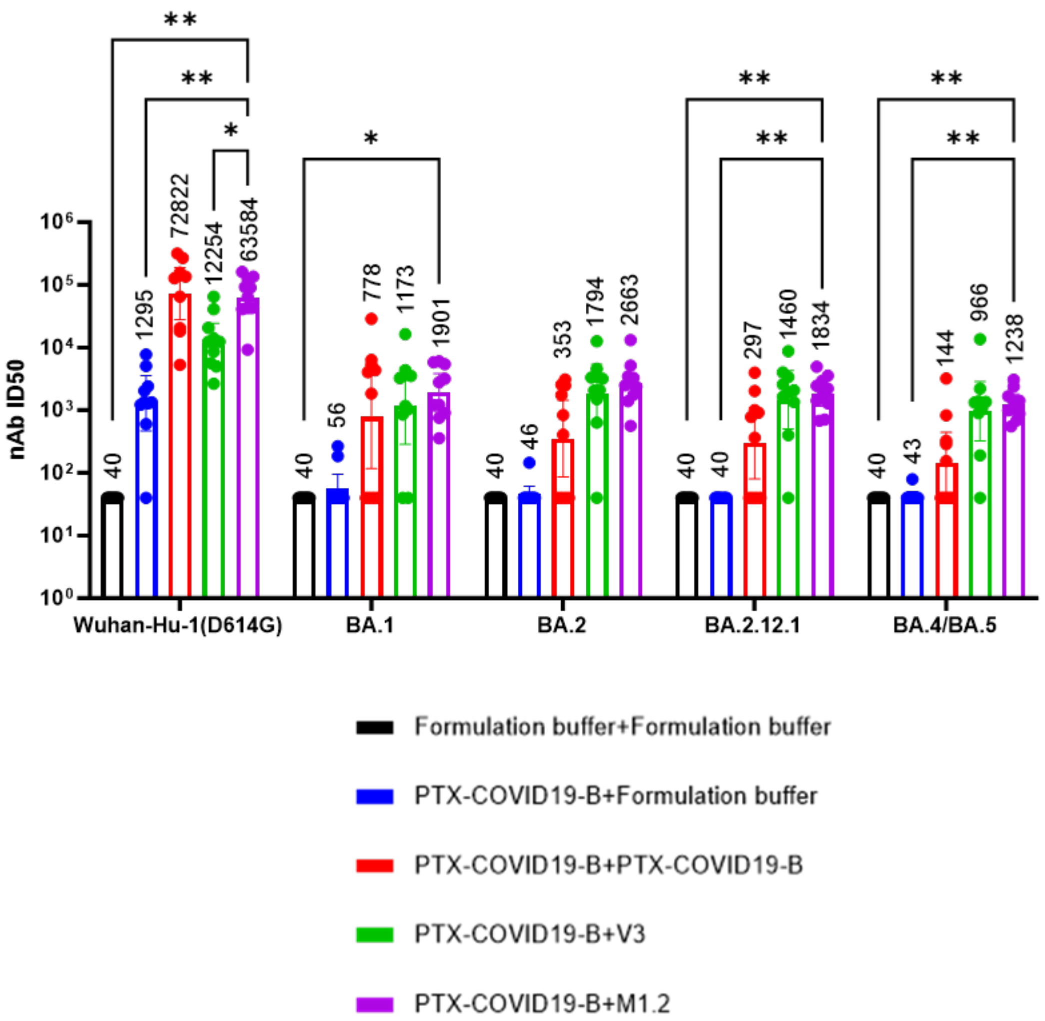

Titers of nAbs in mice sera from the mice shown in Fig. 1 (B). Sera were collected at 4 DPI. Individual mouse serum was used in nAb pseudovirus assay. Columns and error bars indicate geometric mean±95% confidence interval of nAb ID_{50} (n=10). Numbers above column indicate geometric mean values of the nAb ID_{50}. Each dot represents an individual mouse. Two-way ANOVA followed by Tukey's multiple comparison was used for statistical analysis. *: $P<0.05$; **: $P<0.01$.

Supplementary Figure 3

A.

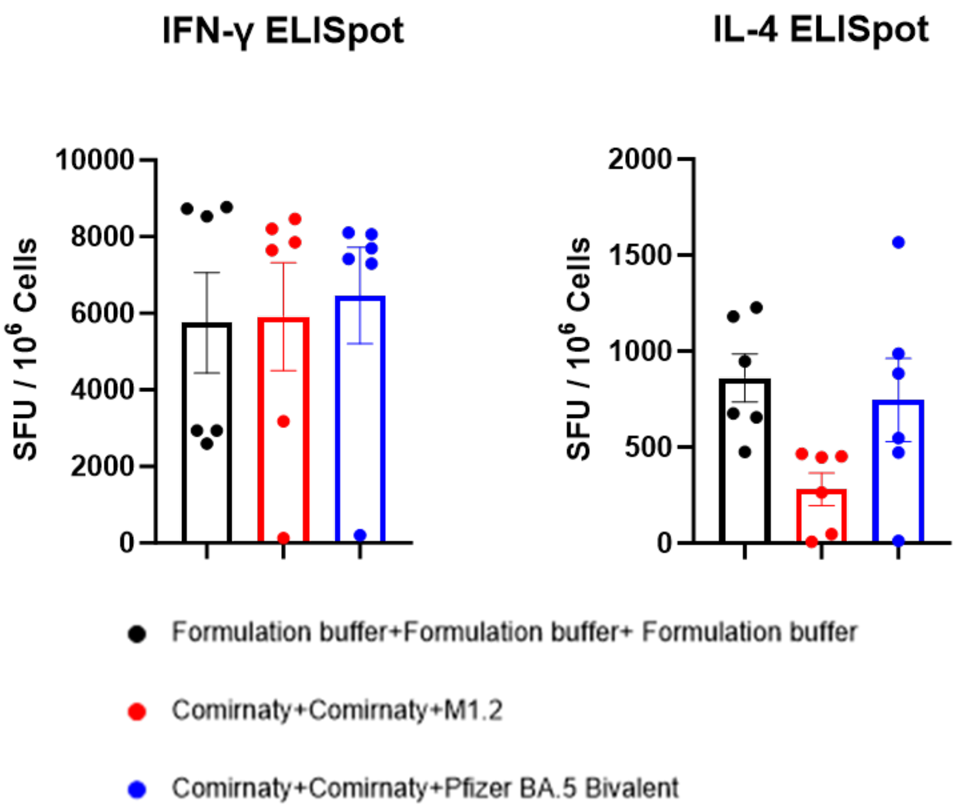

B.

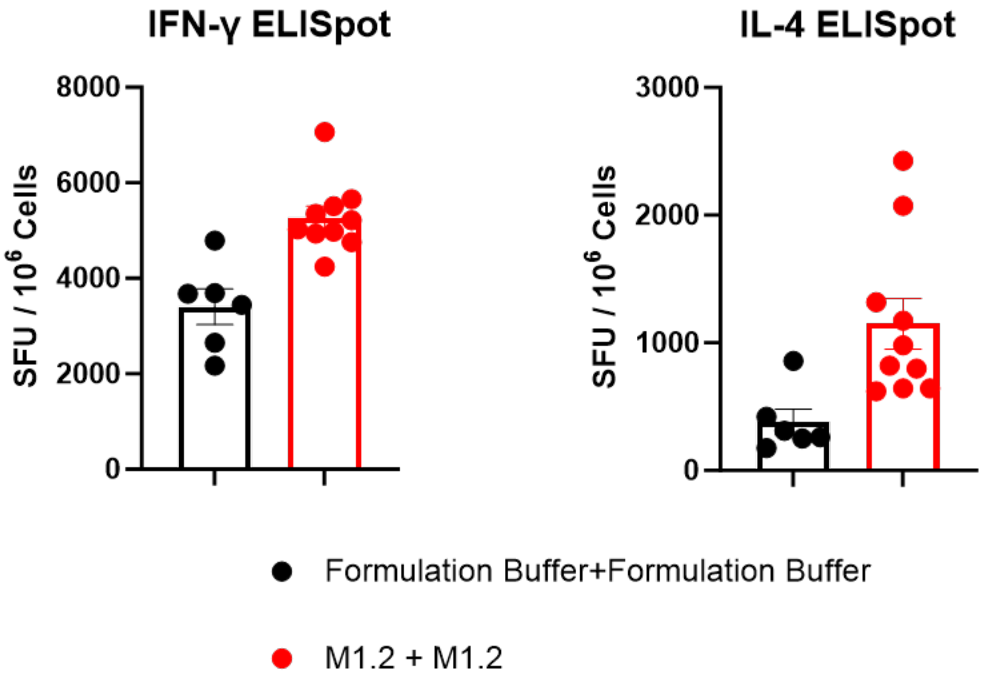

ELISpot results of the mouse splenocytes treated with PMA plus Ionomycin or ConA. (A) Splenocytes in Fig. 3 (D) were stimulated with PMA plus Ionomycin (for IFN-γ) or ConA (for IL-4) as positive control for the ELISpot assay. **(B)** Splenocytes in Fig. 6 (B) were stimulated with PMA plus Ionomycin (for IFN-γ) or ConA (for IL-4) as positive control for the ELISpot assay. Columns and error bars indicate mean± S.E.M. of spot forming units (SFU) per million input cells. Each dot represents an individual mouse.

Supplementary Figure 4

Numbers of different amino acids in spike proteins among SARS-CoV-2 ancestral strain and Omicron subvariants

	Wuhan-Hu-1	BA.1	BA.2	BA.2.12.1	BA.4/BA.5	XBB.1.5	JN.1
BA.1	39	0	28	29	27	36	45
BA.2	30	28	0	3	7	16	35
BA.2.12.1	33	29	3	0	7	18	29
BA.4/BA.5	35	27	7	7	0	19	31
XBB.1.5	42	36	16	18	19	0	36
JN.1	64	45	35	29	31	36	0

Spike protein mutations in Omicron subvariants including JN.1. The number of different amino acids of the pair-wise compared Wuhan-Hu-1 and Omicron subvariant spikes are shown.

Supplementary Table 1: Geometric mean ratio of nAb ID_{50} titers in vaccinated mice sera at 4 DPI

Vaccine regimens #	Pseudoviruses				
	Wuhan-Hu-1 (D614G)	BA.1	BA.2	BA.2.12.1	BA.4/BA.5
PTX-COVID19-B + PTX-COVID19-B *	1	1	1	1	1
PTX-COVID19-B + Formulation buffer	0.018	0.07	0.13	0.13	0.30
PTX-COVID19-B + V3	0.17	1.50	5.08	4.91	6.69
PTX-COVID19-B + M1.2	0.87	2.44	7.54	6.16	8.57

#: Prime vaccine + boost vaccine. *: Reference group.
